# Direct Z-scheme ZnI_2_/GeTe van der Waals heterostructure for photocatalytic overall water splitting

**DOI:** 10.1039/d6ra06043a

**Published:** 2026-09-04

**Authors:** Muhmmad Kashif, Aamir Shahzad, Ateeq-ur -Rehman

**Affiliations:** a Physics Department, Govt. College University Faisalabad 38000 Pakistan mkashif@gcuf.edu.pk; b Physics Department, University of Agriculture Faisalabad 38040 Pakistan ateeq.rehman@uaf.edu.pk

## Abstract

The rising global energy crisis and environmental degradation highlight the urgent demand for efficient photocatalytic materials for solar-driven hydrogen production through water splitting. Two-dimensional (2D) heterostructures are attractive because interfacial coupling can combine efficient charge management with strong redox capability. In this study, first-principles calculations are used to investigate the structural, electronic, optical, and photocatalytic properties of a ZnI_2_/GeTe van der Waals heterostructure. The optimized heterostructure exhibits indirect band gaps of 1.23 eV at the PBE level and 1.96 eV at the HSE06 level, while its equilibrium projected electronic structure has a staggered type-II alignment. Importantly, the charge-transfer mechanism is not assigned from this static band alignment alone. The combined charge-density difference, Bader charge transfer (∼0.037|*e*| from GeTe to ZnI_2_), work-function/electrostatic-potential analysis, and band-decomposed charge densities support a direct Z-scheme-like photoinduced pathway in which the comparatively weaker ZnI_2_-derived conduction electrons recombine with GeTe-derived valence holes at the interface, while strongly reducing electrons are retained in GeTe-derived conduction states for HER and strongly oxidizing holes remain in ZnI_2_-derived valence states for OER. The CHE analysis gives an OER overpotential of approximately 0.70 V at pH = 0 and H-adsorption free energies of 0.360–0.453 eV for the examined HER configurations. The calculated direct Z-scheme solar-to-hydrogen efficiency is 1.18%. These results identify ZnI_2_/GeTe as a promising theoretical platform for photocatalytic overall water splitting while providing a conservative interpretation of the charge-transfer mechanism and solar-energy conversion performance.

## Abstract 2

The rising global energy crisis and environmental degradation highlight the urgent demand for efficient photocatalytic materials for solar-driven hydrogen production through water splitting.

## Graphical abstract

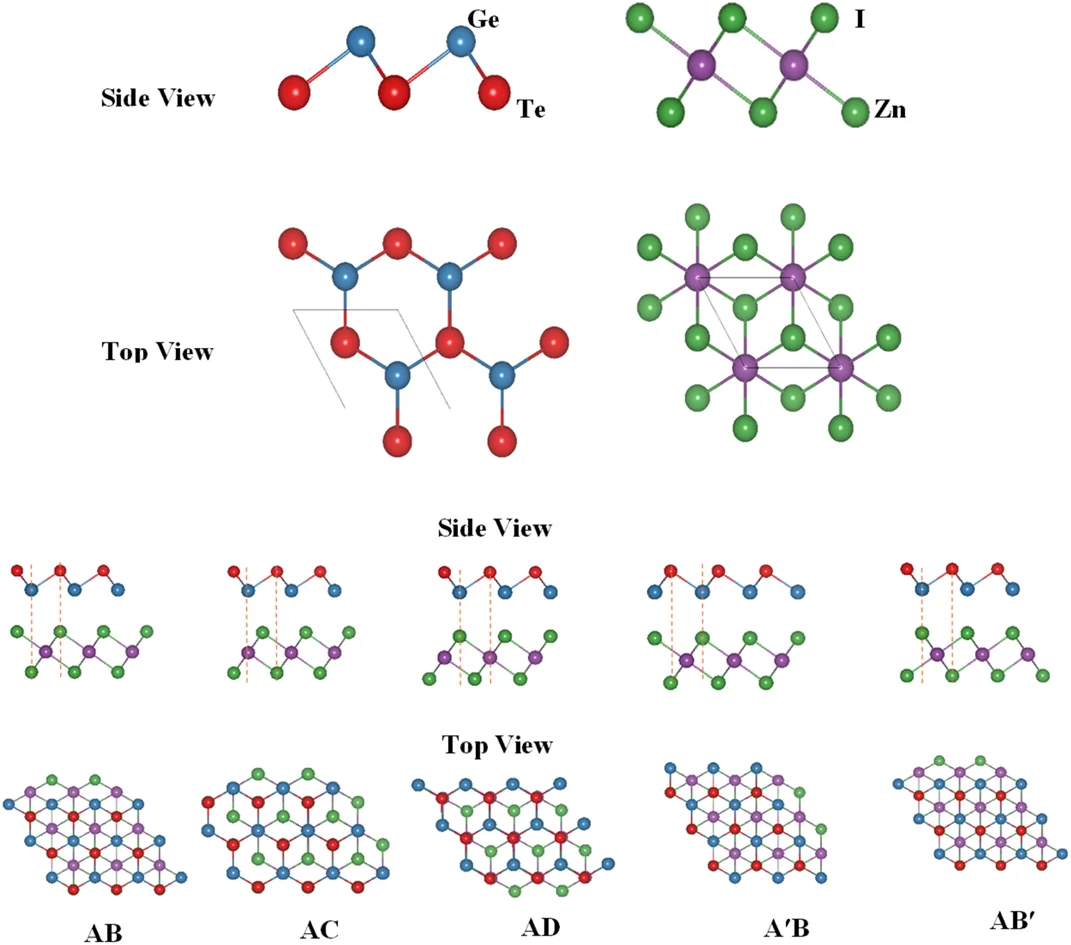

## 1 Introduction

The growing global energy demand and severe environmental concerns associated with the excessive consumption of fossil fuels have accelerated the search for clean, sustainable, and renewable energy sources.^1,2^ Among the available alternatives, hydrogen (H_2_) has attracted significant attention owing to its high energy density, zero carbon emissions, and potential for sustainable energy conversion.^3^ Photocatalytic water splitting is considered a promising approach for hydrogen production because it directly utilizes solar energy to drive redox reactions, offering an environmentally friendly and cost-effective pathway for renewable hydrogen generation.^4–8^ For efficient photocatalytic water splitting, a photocatalyst must satisfy several fundamental criteria.^9^ First, the band gap should exceed 1.23 eV to thermodynamically drive the overall water-splitting reaction. Second, the conduction band minimum (CBM) must be positioned above the reduction potential of H^+^/H_2_, while the valence band maximum (VBM) should lie below the oxidation potential of O_2_/H_2_O. Third, the photocatalyst should effectively absorb a broad portion of the solar spectrum, particularly in the visible region, to maximize solar energy utilization.^10^ In addition, efficient separation and transport of photogenerated charge carriers are crucial to suppress recombination and enhance the hydrogen evolution reaction (HER) and oxygen evolution reaction (OER) kinetics. Two-dimensional (2D) materials have emerged as promising photocatalysts due to their large specific surface area, abundant active sites, tunable electronic properties, and high carrier mobility, which collectively improve light absorption and charge separation efficiency.^11–13^ Moreover, the properties of 2D materials can be further engineered through external strain, defect introduction, doping, surface adsorption, and heterostructure construction, enabling flexible optimization for photocatalytic applications.^14,15^ However, in isolated 2D monolayer photocatalysts, photogenerated electrons and holes are typically generated on the same surface, which leads to rapid carrier recombination and limits photocatalytic efficiency. To overcome this limitation, van der Waals (vdW) heterostructures composed of vertically stacked 2D monolayers have been proposed as an effective strategy to spatially separate charge carriers and enhance photocatalytic performance.^16^ In conventional type-II heterostructures, electrons and holes migrate to different layers due to staggered band alignment, resulting in prolonged carrier lifetimes. Numerous type-II vdW heterostructures, such as GeSe/AsP,^17^ SnC/SnSSe,^18^ BAs/CrS_2,_^19^ Zr_2_CO_2_/WS_2,_^20^ MoSeO/BP,^21^ and ZrS_2_/PtS_2,_^22^ have demonstrated promising photocatalytic properties. However, the reduced redox potential of charge carriers in type-II systems often limits their overall water-splitting capability.^23^ In contrast, Z-scheme heterostructures offer a superior charge transfer mechanism that combines efficient carrier separation with strong redox ability.^24–26^ In a direct Z-scheme system, photogenerated electrons in the conduction band of one semiconductor recombine with holes in the valence band of the other at the interface, while highly energetic electrons and holes are retained on separate layers for HER and OER, respectively.^27^ This charge transfer pathway not only suppresses bulk recombination but also preserves strong oxidation and reduction potentials, which are essential for overall water splitting. Consequently, direct Z-scheme vdW heterostructures have attracted increasing interest for high-efficiency solar-to-hydrogen conversion. Germanium telluride (GeTe) monolayers have been reported to possess suitable band edge positions, high carrier mobility, and strong optical absorption in the visible region, making them promising candidates for photocatalytic hydrogen evolution.^28^ Similarly, two-dimensional ZnI_2_ monolayers exhibit appropriate band edge alignment for both HER and OER and show tunable electronic and optical properties under strain.^29^ These complementary properties suggest that combining ZnI_2_ and GeTe into a vdW heterostructure could yield a highly efficient photocatalyst with enhanced charge separation and redox capability.

Despite the individual advantages of ZnI_2_ and GeTe monolayers, to the best of our knowledge, the ZnI_2_/GeTe vdW heterostructure has not previously been investigated for photocatalytic water splitting. Here, we perform a comprehensive first-principles study of its structural, mechanical, electronic, optical, interfacial, and photocatalytic properties. In addition to binding-energy, phonon, and AIMD analyses, the lattice-mismatch accommodation is quantified explicitly through the common lattice constant, residual strain of each constituent, and strain energy. The photoinduced charge-transfer mechanism is assessed by combining the projected electronic structure, vacuum-referenced band alignment, charge-density difference, Bader analysis, work-function mismatch, planar-averaged electrostatic potential, and band-decomposed charge densities rather than relying on staggered band alignment alone. Carrier transport, HER/OER thermodynamics, optical absorption, and STH efficiency are then evaluated using the consistent procedures described below. The combined results support a direct Z-scheme-like photocatalytic pathway and provide a quantitative assessment of the structural and energetic assumptions entering the ZnI_2_/GeTe model.

## 2 Computational methodology

First-principles calculations were performed within density functional theory (DFT) using the Vienna *ab initio* Simulation Package (VASP).^30,31^ The interaction between ion cores and valence electrons was described by the projector augmented-wave (PAW) method, and exchange-correlation effects were treated using the Perdew–Burke–Ernzerhof (PBE) generalized-gradient approximation.^32^ Long-range van der Waals interactions were included using the DFT-D3 correction.^33^ A plane-wave cutoff energy of 520 eV and an 11 × 11 × 1 Monkhorst–Pack *k*-point mesh were used, with structural relaxation continued until the total-energy and residual-force criteria reached 10^−6^ eV and 0.01 eV Å^−1^, respectively. A 25 Å vacuum region and dipole correction were applied along the out-of-plane direction. HSE06 calculations with 25% exact exchange were used for the improved band-gap and band-edge description.^34^ Dynamic stability was evaluated by finite-displacement phonon calculations using PHONOPY. Thermal stability was examined by *ab initio* molecular dynamics (AIMD) using a 3 × 3 ZnI_2_/GeTe supercell (45 atoms) in the canonical NVT ensemble at 300 K with a 1 fs time step and for 8.0 ps. Charge redistribution was analyzed using charge-density differences and Bader charge analysis, while planar-averaged electrostatic potentials provided vacuum references, work functions, and the interfacial potential step. Carrier mobility was evaluated within deformation-potential theory at 300 K,^35^ using the symmetric strain sequence −0.5%, −0.25%, 0, +0.25%, and +0.5%; the strained band edges were referenced to their corresponding vacuum levels and effective masses were evaluated at the actual carrier extrema. HER and OER thermodynamics were treated using the computational hydrogen electrode (CHE) framework at 298.15 K.^36^ Surface-reaction calculations used a 2 × 2 ZnI_2_/GeTe supercell with one adsorbate or reaction intermediate per supercell and included zero-point-energy and entropy corrections. Optical absorption was obtained from the frequency-dependent dielectric response. The solar-to-hydrogen (STH) efficiency was evaluated using the direct Z-scheme formalism implemented in PySTH,^37^ the AM1.5G spectrum, and HSE06 constituent-layer band gaps; no Janus-specific intrinsic-vacuum-level correction was applied.

For the commensurate 1 × 1 ZnI_2_/GeTe model, a common in-plane lattice constant *a*_HS_ = 3.981 Å was used. The residual strain imposed on constituent *i* was evaluated as1
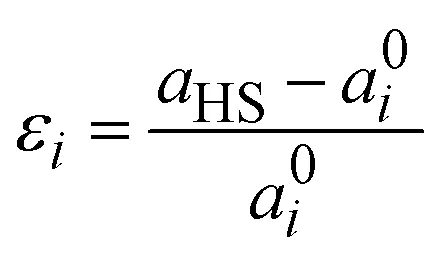
where *_i_*^0^ is the optimized isolated-monolayer lattice constant. The corresponding lattice-accommodation energy was evaluated from

2 *E*_str,*i*_ = *E*_*i*_(*a*_HS_) − *E*_*i*_(*a*^0^_*i*_)

Binding energies of all stacking configurations were referenced to fully relaxed isolated monolayers under the same computational settings, so the reported net binding energies include the small strain-energy contribution associated with lattice matching. For interfacial electronic analysis, band edges were placed on a common vacuum reference obtained from the planar-averaged electrostatic potential and work functions were defined using the standard positive convention *Φ* = *E*_vac_ − *E*_F_

Band-decomposed charge densities were calculated for the relevant valence- and conduction-band states to examine their layer localization and interfacial weight.

ChatGPT (OpenAI) was used during manuscript preparation with prompts requesting language refinement, improvement of scientific clarity and organization, and assistance in presenting author-provided computational results. The tool was not used to generate the computational data or research results. All AI-assisted content was reviewed, edited, and verified by the authors.

## 3 Results and discussion

### 3.1 Structure and stability

The optimized atomic structures of the ZnI_2_ and GeTe monolayers are shown in Fig. 1, including both top and side views. The ZnI_2_ monolayer adopts a hexagonal lattice with an optimized lattice constant of *a* = 4.03 Å. The calculated Zn–I bond length is 2.84 Å, which is in good agreement with previously reported theoretical results.^29,38^ Similarly, the GeTe monolayer exhibits a hexagonal structure with an optimized lattice constant of *a* = 3.90 Å and a Ge–Te bond length of 2.75 Å, consistent with earlier studies.^28,39^

**Fig. 1:**
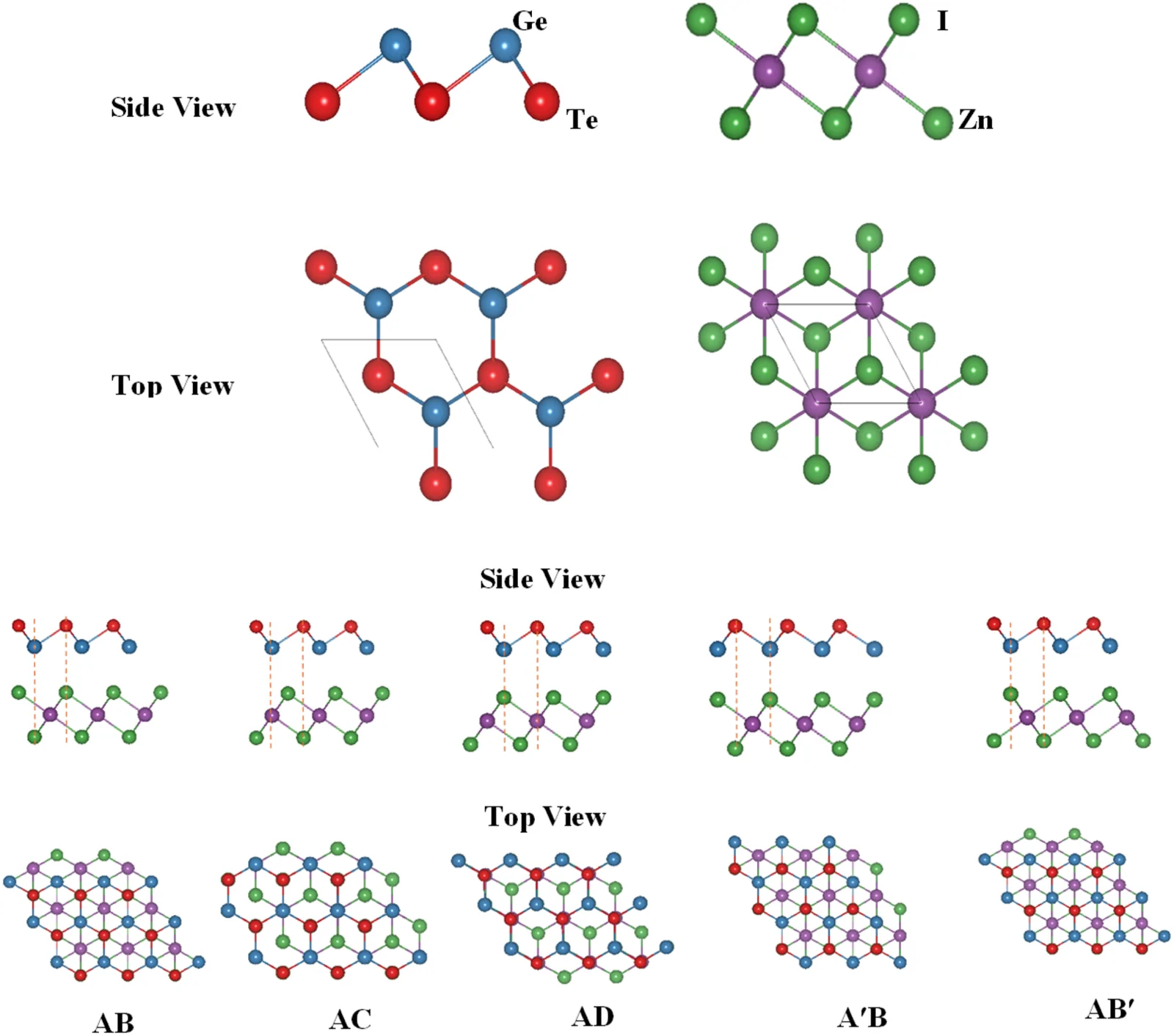
Top and side views of the GeTe and ZnI_2_ monolayers and ZnI_2_/GeTe heterostructure with the five considered stacking patterns (AB, AC, AD, A′B, and AB′).

The optimized isolated lattice constants are 4.03 Å for ZnI_2_ and 3.90 Å for GeTe, corresponding to an initial lattice mismatch of 3.27%. A commensurate 1 × 1 ZnI_2_/GeTe cell with a common in-plane lattice constant *a*_HS_ = 3.981 Å (interfacial area ≈ 13.72 Å^2^) was used to distribute the mismatch between the constituents. Relative to the isolated monolayers, this gives a residual compressive strain of approximately −1.22% in ZnI_2_ and a tensile strain of +2.07% in GeTe. Five relative configurations, AB, AC, AD, AB′, and A′B, were examined, as shown in Fig. 1. The lattice-accommodation energies are 8.09 meV per primitive cell for ZnI_2_ and 11.36 meV per primitive cell for GeTe, giving a total strain energy of 19.45 meV per heterostructure cell, or approximately 1.42 meV Å^−2^. Thus, the common cell is obtained with only a small strain-energy contribution. The relative stability of the five stackings was quantified from the binding energy per unit area.

The binding energy per unit interfacial area was calculated as3
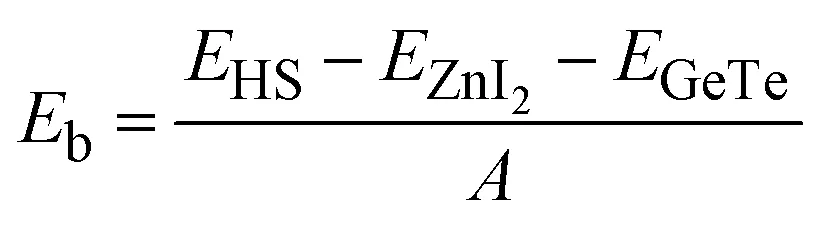
where *E*_HS_, *E*_ZnI_2__, and *E*_GeTe_ are the total energies of the heterostructure and the isolated ZnI_2_ and GeTe monolayers, respectively, and *A* is the interfacial area. The same computational settings were used for the heterostructure and isolated-component reference calculations.

The equilibrium interlayer spacings and net binding energies for AB, AC, AD, AB′, and A′B are (3.40 Å, −14.91 meV Å^−2^), (≈3.43 Å, −13.98 meV Å^−2^), (≈4.05 Å, −9.35 meV Å^−2^), (≈4.08 Å, −9.11 meV Å^−2^), and (≈4.09 Å, −9.11 meV Å^−2^), respectively. All five configurations are energetically favorable when referenced to the fully relaxed isolated monolayers. AB has the shortest equilibrium separation and the most negative binding energy and is lower in total energy than AC, AD, AB′, and A′B by approximately 12.79, 76.30, 79.66, and 79.66 meV per primitive cell, respectively. The AB configuration was therefore used for subsequent calculations. Its binding strength is of the same order as stable vdW heterostructures such as HfSSe/arsenene and ZnS/SnS_2_ (Fig. 2).^40,41^

**Fig. 2:**
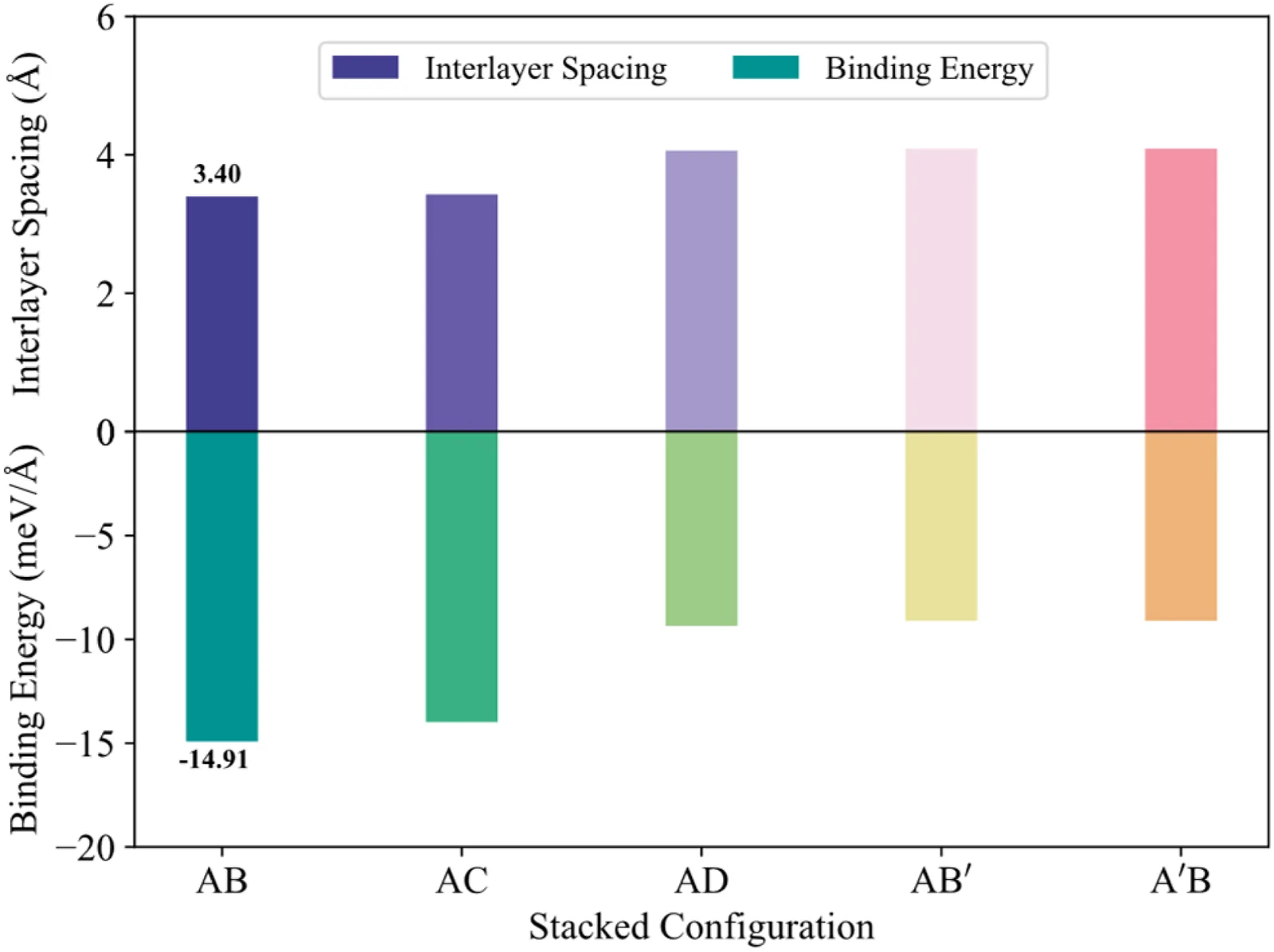
Equilibrium interlayer spacing and binding energy of the five ZnI_2_/GeTe stacking configurations.

Dynamic stability was evaluated from the phonon dispersion shown in Fig. 3(A). No significant imaginary branches occur across the Brillouin zone; the very small negative frequencies near *Γ* are treated as the usual numerical sensitivity of long-wavelength flexural modes in two-dimensional phonon calculations rather than evidence of structural instability.^42^

**Fig. 3:**
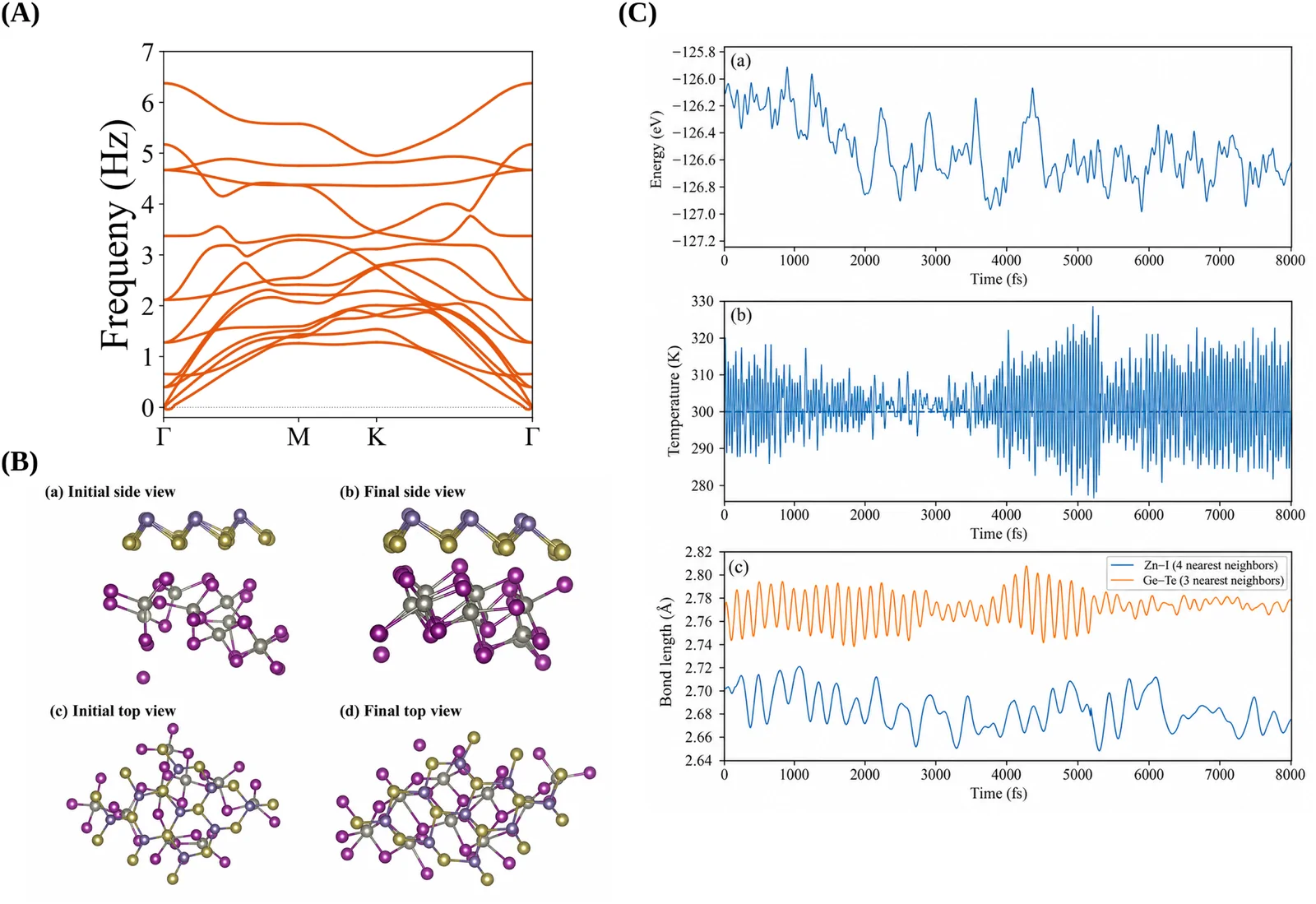
Structural-stability assessment of ZnI_2_/GeTe: (A) phonon dispersion, (B) initial and final AIMD structures, and (C) temperature, energy, and representative bond-length evolution during the 8 ps AIMD simulation at 300 K.

Finite-temperature structural integrity was further assessed from the AIMD trajectory at 300 K see Fig. 3(C). The analyzed 8.0 ps simulation gives a mean temperature of 299.96 ± 8.35 K (range 274–329 K), while the energy trace remains bounded over the simulation window. Quantitative bond tracking yields a trajectory-averaged four-nearest-neighbor Zn–I distance of 2.6820 ± 0.0156 Å and a three-nearest-neighbor Ge–Te distance of 2.7708 ± 0.0119 Å. The corresponding values change from 2.7048 to 2.6814 Å for Zn–I and from 2.7586 to 2.7760 Å for Ge–Te between the first production configuration and 8 ps. At the end of the trajectory, all nine Zn sites retain at least four I neighbors and all nine Ge sites retain three Te neighbors within a 3.2 Å cutoff. The shortest interlayer I–Te separation averages 3.5936 ± 0.0928 Å and never falls below 3.3011 Å. Thus, no persistent bond breaking or interfacial reconstruction is observed, supporting retention of the heterostructure framework at 300 K over the analyzed production window.

### 3.2 Mechanical properties

The mechanical stability and elastic properties of the ZnI_2_ and GeTe monolayers, as well as the ZnI_2_/GeTe heterostructure, were evaluated from their in-plane elastic constants. Owing to the hexagonal symmetry of the monolayers and heterostructure, the independent elastic constants C_11_, C_12_, and C_66_ were determined. The calculated elastic parameters are summarized in Table 1.

**Table 1:** Calculated in-plane elastic constants *C*_11_ and *C*_12_, shear modulus *G*, two-dimensional Young's modulus *Y*^D^_2_, Poisson's ratio *ν*^D^_2_, and mechanical-stability status

System	*C*_11_ (N m^−1^)	*C*_12_ (N m^−1^)	*G* (N m^−1^)	*Y* ^D^_2_ (N m^−1^)	*ν* ^D^_2_	Status
ZnI_2_	43.05	18.68	12.18	34.94	0.43	Stable
GeTe	37.40	8.20	14.59	35.60	0.22	Stable
ZnI_2_/GeTe	78.58	26.79	25.89	69.45	0.34	Stable

The mechanical stability of two-dimensional materials can be assessed using the Born–Huang stability criteria, which require *C*_11_ > 0, *C*_66_ > 0, and (*C*_11_–*C*_12_) > 0. All calculated elastic constants for the ZnI_2_ and GeTe monolayers and the ZnI_2_/GeTe heterostructure satisfy these conditions, confirming their mechanical stability. The two-dimensional Young's modulus (*Y*^D^_2_) reflects the in-plane stiffness of the material, while Poisson's ratio (*ν*^D^_2_) characterizes the transverse mechanical response under applied strain. As listed in Table 1, the ZnI_2_/GeTe heterostructure exhibits a significantly higher Young's modulus compared to the individual monolayers, indicating enhanced mechanical rigidity upon heterostructure formation. The calculated Poisson's ratios for all systems fall within the typical range of 0–0.5 for stable 2D materials, further confirming their mechanical robustness. To investigate the mechanical anisotropy, the angular dependence of the Young's modulus, shear modulus, and Poisson's ratio was analyzed. The corresponding polar plots for the ZnI_2_/GeTe heterostructure are provided in Fig. S1 of the (SI). The nearly circular shapes of these plots indicate that the heterostructure exhibits an almost isotropic mechanical response in the basal plane. Such isotropic mechanical behavior is advantageous for practical applications, as it ensures uniform mechanical performance under strain applied along different in-plane directions.

### 3.3 Electronic properties

The electronic band structures of the ZnI_2_ and GeTe monolayers, as well as the ZnI_2_/GeTe heterostructure, were calculated using both the PBE and HSE06 exchange–correlation functionals and are shown in Fig. 4. The use of the hybrid HSE06 functional provides a more accurate description of the band gaps and band edge positions, which is essential for evaluating photocatalytic water-splitting performance.

**Fig. 4:**
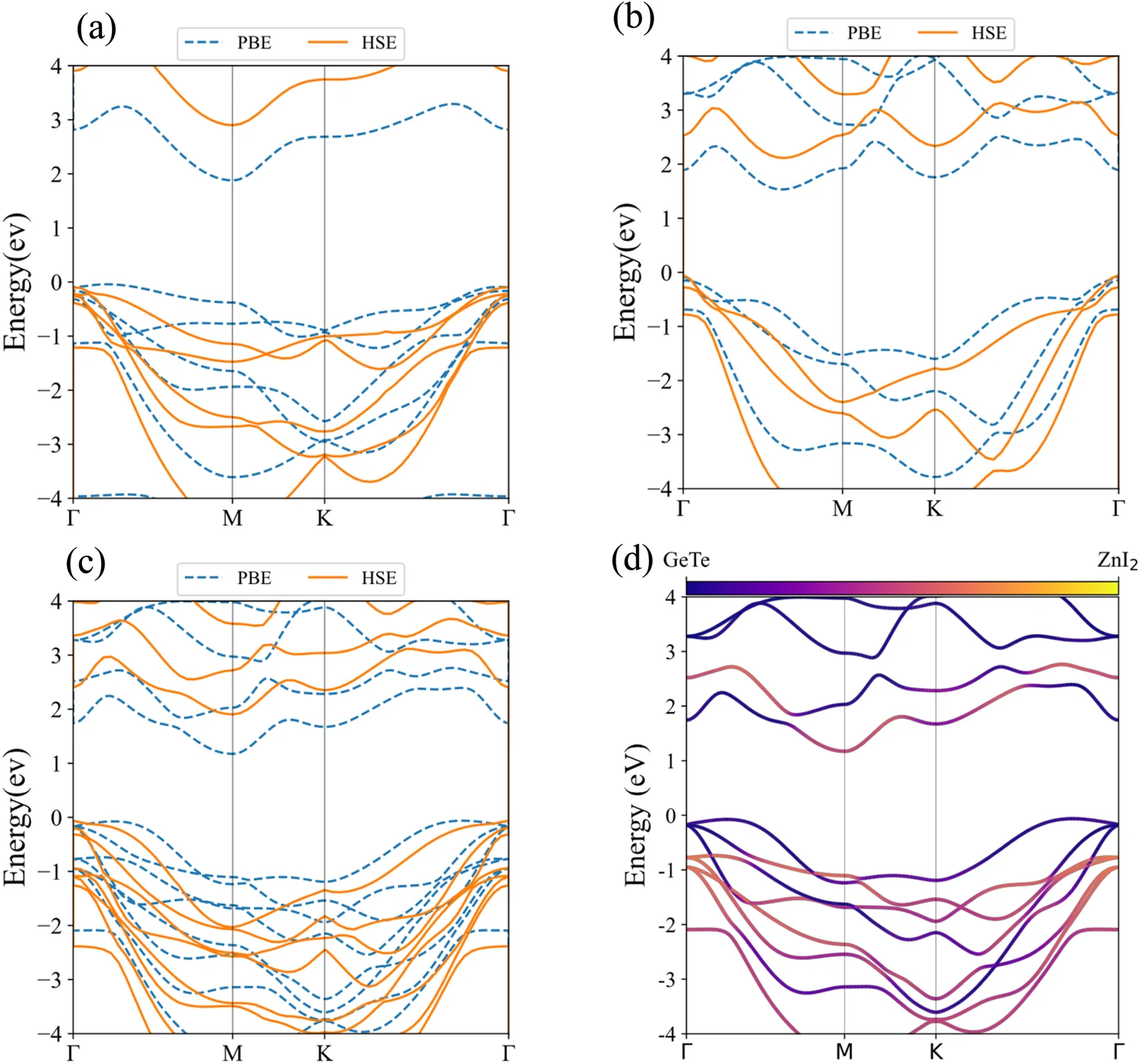
Band structures of (a) ZnI_2_, (b) GeTe, and (c) ZnI_2_/GeTe, together with (d) the projected band structure of the heterostructure.

The ZnI_2_ monolayer exhibits an indirect band gap of 1.92 eV at the PBE level and 2.99 eV at the HSE06 level,^29^ whereas GeTe has corresponding gaps of 1.61 and 2.16 eV.^43^ The assembled ZnI_2_/GeTe heterostructure retains an indirect gap of 1.23 eV at the PBE level and 1.96 eV at the HSE06 level. The projected band structure in Fig. 4(d) shows that the global CBM of the equilibrium heterostructure is predominantly ZnI_2_-derived, whereas the global VBM is mainly GeTe-derived; the PDOS/TDOS analysis in Fig. 5 is consistent with this layer-resolved character. This is a staggered type-II alignment in the static equilibrium electronic structure. However, a static type-II alignment does not by itself determine the photoinduced charge-transfer pathway. A conventional type-II process would retain electrons at the lower-energy ZnI_2_ conduction edge and holes at the GeTe valence edge, thereby sacrificing part of the reduction and oxidation power. The direct Z-scheme-like assignment is therefore evaluated below from the combined interfacial electrostatics and state-resolved charge-density evidence rather than inferred from the band alignment alone.

**Fig. 5:**
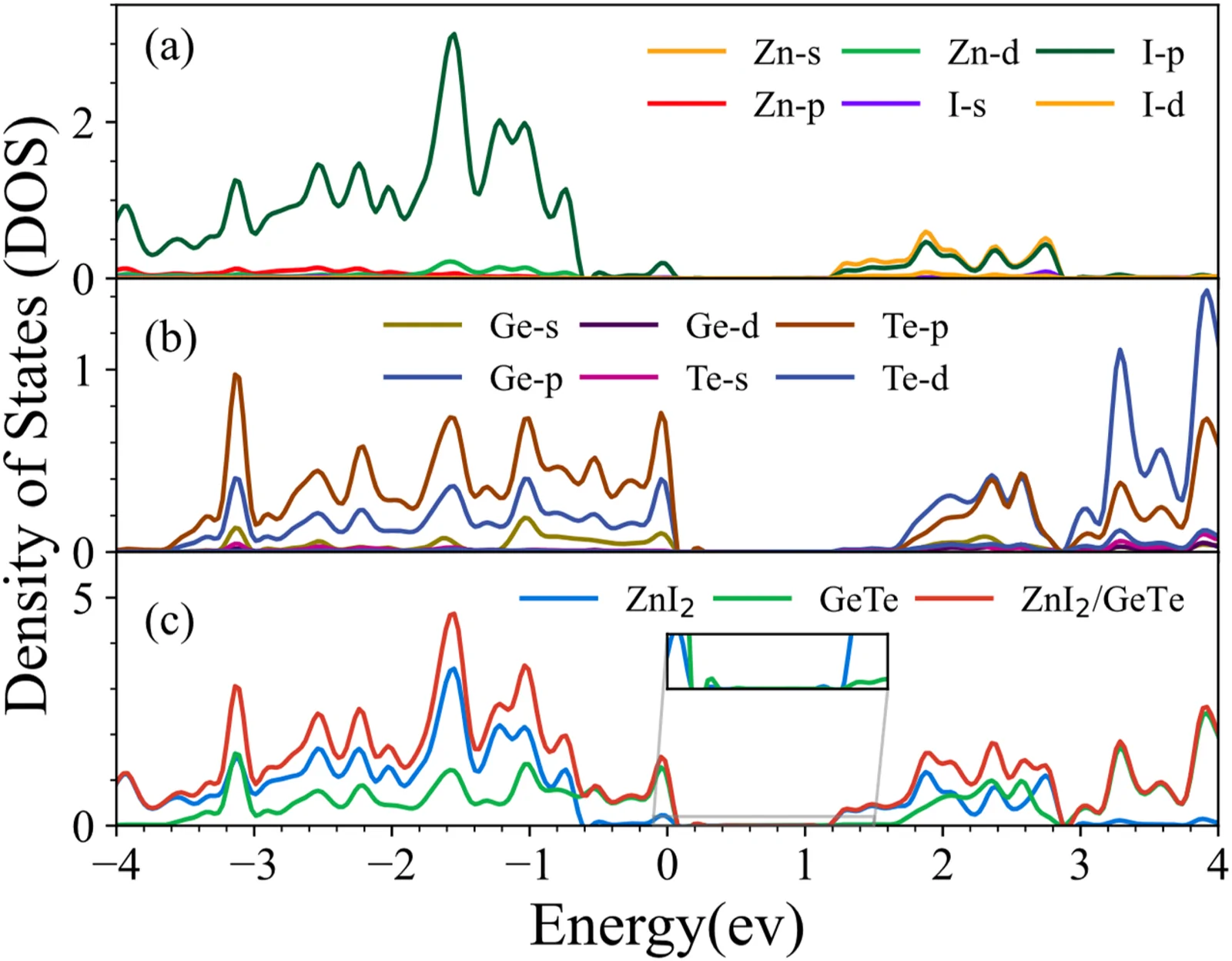
Projected density of states of (a) ZnI_2_ and (b) GeTe, and (c) total density of states of ZnI_2_, GeTe, and ZnI_2_/GeTe.

### 3.4 Interfacial charge redistribution and band alignment

Charge redistribution at the interface plays a crucial role in determining the internal electric field and charge transfer behavior of van der Waals heterostructures. To investigate the interfacial charge transfer in the ZnI_2_/GeTe heterostructure, the charge density difference was calculated using the following expression:

4 Δ*ρ* = *ρ*_ZnI_2_/GeTe_ − *ρ*_ZnI_2__ − *ρ*_GeTe_

where *ρ*_ZnI_2__/GeTe, *ρ*_ZnI_2__, and *ρ*_GeTe_ denote the charge densities of the heterostructure, ZnI_2_ monolayer, and GeTe monolayer, respectively.

The planar-averaged and three-dimensional charge-density differences in Fig. 6(A) show clear ground-state charge redistribution after interface formation. Charge accumulation occurs predominantly toward the ZnI_2_ side, whereas depletion is concentrated toward GeTe, indicating equilibrium electron transfer from GeTe to ZnI_2_. This redistribution creates an interfacial dipole and a built-in electrostatic field directed from GeTe toward ZnI_2_. The charge-density difference therefore establishes the equilibrium transfer direction and interfacial electrostatic environment; it is not, by itself, direct evidence of the photoexcited carrier pathway. Bader analysis gives a net equilibrium transfer of approximately 0.037|*e*| from GeTe to ZnI_2_. Using the standard positive work-function definition *Φ* = *E*_vac_ − *E*_F_, the isolated ZnI_2_ and GeTe monolayers have work functions of approximately 6.57 and 5.38 eV, respectively. The lower work function of GeTe is consistent with electron redistribution toward ZnI_2_ upon contact. After contact, the two sides show work-function magnitudes of approximately 5.60 and 5.33 eV, while the planar-averaged electrostatic potential in Fig. 6(B) gives an interfacial vacuum-level step Δ*Φ* ≈ 0.27 eV.

**Fig. 6:**
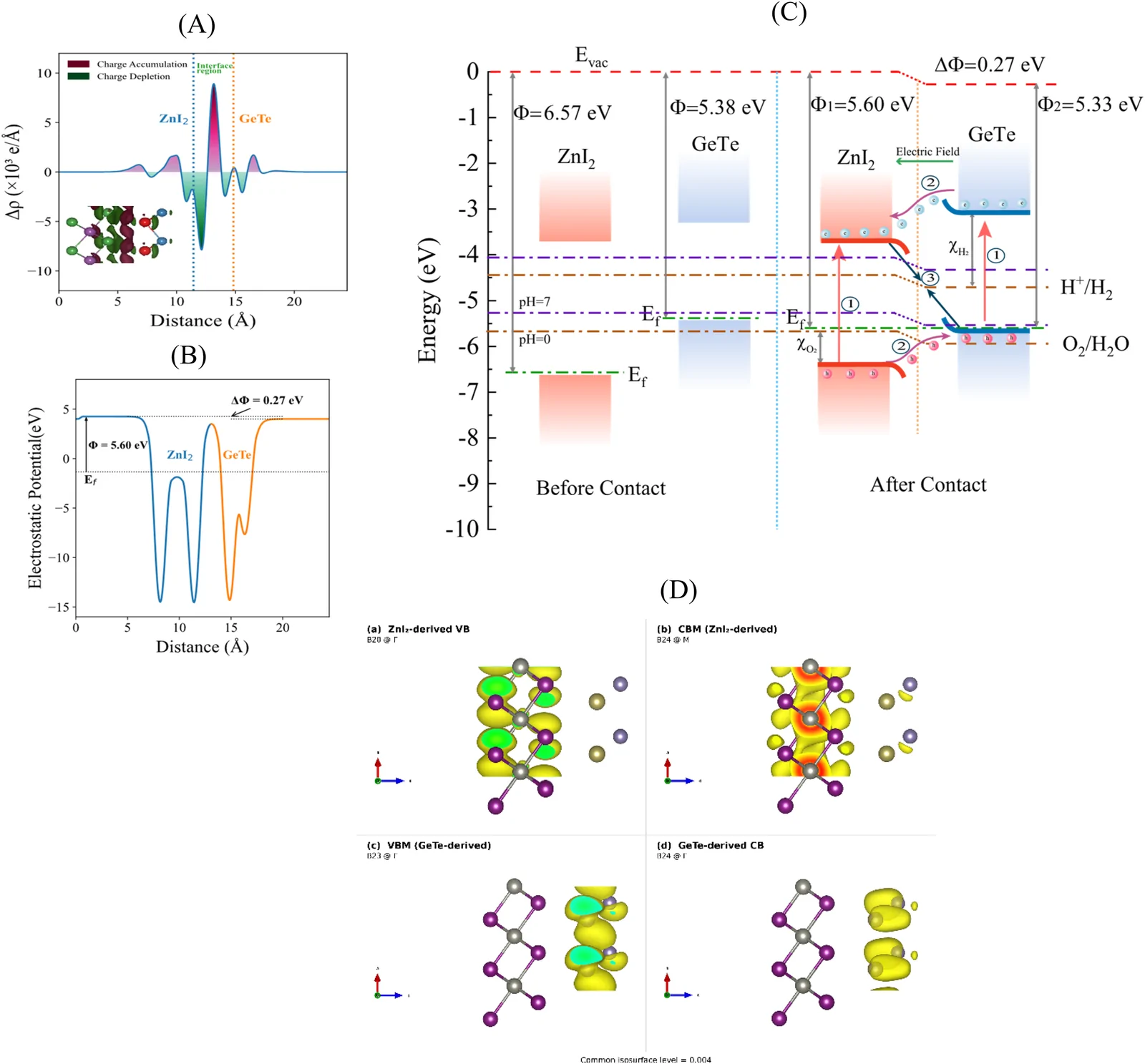
Interfacial electronic structure and evidence for the proposed Z-scheme-like pathway in ZnI_2_/GeTe: (A) ground-state-density difference, (B) planar-averaged electrostatic potential with Δ*Φ* ≈ 0.27 eV, (C) vacuum-referenced band alignment and proposed charge-transfer pathway, and (D) band-decomposed charge densities of the relevant ZnI_2_- and GeTe-derived valence and conduction states (common isosurface = 0.004).

The vacuum-referenced band alignment in Fig. 6(C) provides the numerical levels used for the redox analysis. Before contact, the isolated ZnI_2_ CBM/VBM are approximately −3.67/−6.66 eV and the GeTe CBM/VBM are approximately −3.26/−5.43 eV. In the contacted heterostructure diagram, the layer-resolved ZnI_2_ CBM/VBM are approximately −3.69/−6.54 eV, whereas the GeTe CBM/VBM are approximately −3.20/−5.66 eV. The corresponding conduction- and valence-band offsets are about 0.49 and 0.88 eV, respectively. At pH = 0, the retained GeTe-derived conduction states provide a HER redox driving force *χ*(H_2_) ≈ 1.24 eV and the retained ZnI_2_-derived valence states provide an OER driving force *χ*(O_2_) ≈ 0.87 eV. These band-edge driving forces are distinct from the surface-reaction OER overpotential and H-adsorption free energy discussed in Section 3.6.

The water redox reference levels shift with pH according to the Nernst relation. The standard vacuum-referenced reduction and oxidation levels used for comparison are expressed below as functions of pH:

5 H^+^/H_2_ = − 4.44 eV + pH × 0.059 eV

6 O_2_/H_2_O = − 5.67 eV + pH × 0.059 eV

At pH = 7, the water redox levels shift upward on the vacuum scale according to the Nernst term, whereas the intrinsic electronic band structure of the solid is not assumed to undergo the same rigid shift. Fig. 6(C) should therefore be interpreted as a thermodynamic band-edge criterion at the specified pH rather than as evidence that all elementary HER/OER steps become spontaneous. Surface-reaction thermodynamics are treated separately and consistently within the CHE framework in Section 3.6.

Taken together, the interfacial and band-resolved results support a direct Z-scheme-like photoinduced charge-transfer pathway rather than a simple conventional type-II transfer. Upon illumination, electron–hole pairs are generated primarily within the two semiconductor constituents. The work-function mismatch, equilibrium GeTe → ZnI_2_ charge transfer, interfacial potential step, and built-in field establish an electrostatic environment favorable for selective interfacial recombination. The band-decomposed charge densities in Fig. 6(D) show the spatial distributions of the relevant ZnI_2_- and GeTe-derived conduction and valence states. The ZnI_2_-derived conduction state and GeTe-derived valence state show interfacial proximity/weight consistent with the proposed recombination channel, whereas the complementary GeTe-derived conduction states and ZnI_2_-derived valence states remain associated with the reduction and oxidation sides and retain the stronger redox capability required for HER and OER. This state selectivity distinguishes the proposed pathway from conventional type-II transfer. Because the evidence is based on static electronic-structure calculations rather than time-resolved excited-state dynamics, it supports, but does not directly prove, the proposed Z-scheme-like mechanism.^40,41,44,45^

### 3.5 Carrier mobility

Carrier transport was evaluated within deformation-potential theory at 300 K using a consistent dataset for ZnI_2_, GeTe, and ZnI_2_/GeTe. Uniaxial strains were applied independently along the two in-plane lattice-vector directions, denoted *x* and *y*. A symmetric strain sequence of −0.5%, −0.25%, 0, +0.25%, and +0.5% was used to determine the deformation-potential constants and in-plane elastic moduli. To avoid artificial reference shifts, the CBM and VBM energies of each strained structure were referenced to the corresponding vacuum level. Effective masses were evaluated from the local band curvature at the actual carrier extrema rather than imposing a common high-symmetry point. The numerical extrema, masses, deformation-potential fit quality, and strain-energy/band-edge fits are summarized in the (SI). The carrier mobility along in-plane direction *i* is expressed as7
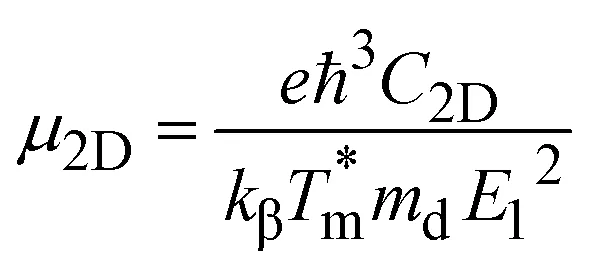
where *e* is the elementary charge, ℏ is the reduced Planck constant, *k*_B_ is the Boltzmann constant, *T* = 300 K, *C*_*i*_ is the two-dimensional elastic modulus along transport direction *i*, 
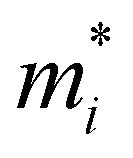
 is the corresponding transport effective mass,
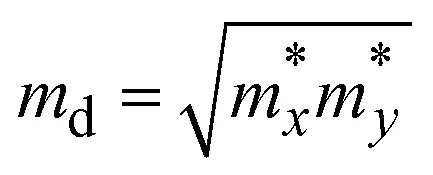
is the density-of-states effective mass, and *E*_1_^*i*^ is the deformation-potential constant.

The elastic modulus was obtained from the quadratic strain-energy relation 8
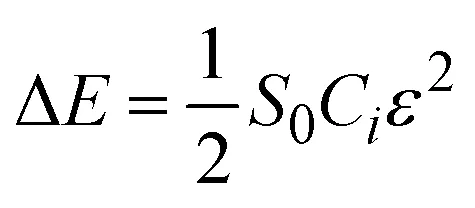
where *S*_0_ is the equilibrium in-plane area and *ε* is the applied uniaxial strain. The deformation-potential constant *E*_1_^*i*^ was determined from the slope of the vacuum-referenced band-edge energy with respect to strain. The corresponding strain-energy and vacuum-referenced band-edge fitting curves, together with the carrier extrema, directional effective masses, and deformation-potential fit quality, are provided in the SI. The key transport parameters and calculated mobilities are summarized in Table 2.

**Table 2:** Directional effective masses (m*), deformation-potential constants (*E*_1_), in-plane elastic moduli (*C*), and carrier mobilities (*µ*) of ZnI_2_, GeTe, and ZnI_2_/GeTe at 300 K

Carrier	System	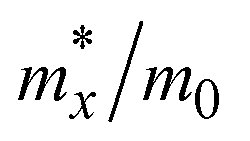	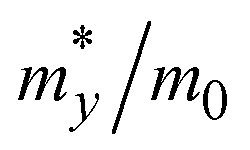	*E*_1_^*x*^ (eV)	*E*_1_^*y*^ (eV)	*C*_*x*_ (N m^−1^)	*C*_*y*_ (N m^−1^)	*µ*_*x*_ (cm^2^ V^−1^ s^−1^)	*µ*_*y*_ (cm^2^ V^−1^ s^−1^)
Electron	ZnI_2_	0.276	0.630	5.89	1.59	35.59	35.63	189.87	1142.67
Electron	GeTe	0.180	0.464	4.86	2.59	31.58	31.60	547.35	748.29
Electron	ZnI_2_/GeTe	0.253	0.640	5.24	2.11	65.95	65.95	502.48	1225.05
Hole	ZnI_2_	0.261	1.681	6.83	2.42	35.59	35.63	94.00	116.38
Hole	GeTe	0.912	0.531	7.28	5.18	31.58	31.60	19.99	67.89
Hole	ZnI_2_/GeTe	1.449	2.357	0.26	1.13	65.95	65.95	7759.28	252.53

As summarized in Table 2, the carrier mobilities exhibit pronounced directional anisotropy despite the nearly isotropic in-plane elastic response. For the ZnI_2_/GeTe heterostructure, the electron mobilities are 502.48 and 1225.05 cm^2^ V^−1^ s^−1^ along the *x* and *y* directions, respectively, while the corresponding hole mobilities are 7759.28 and 252.53 cm^2^ V^−1^ s^−1^. The more favorable electron transport along *y* and the strong directional variation in hole transport therefore arise primarily from the combined effects of band curvature and deformation-potential coupling rather than from the elastic modulus alone. The *y*-direction electron mobility remains of the same order of magnitude as theoretical deformation-potential mobilities reported for other 2D heterostructures.^46–48^

The particularly large *x*-direction hole mobility of the heterostructure originates from the small deformation-potential constant *E*_1x,h_ = 0.26 eV. The corresponding band-edge fit is less linear (*R*^2^ ≈ 0.714) than the other deformation-potential fits; because *µ* varies as *E*_1_^−2^, this mobility is especially sensitive to uncertainty in the fitted slope and should therefore be interpreted cautiously.

The calculated mobilities represent intrinsic estimates within the deformation-potential approximation, which describes acoustic deformation-potential scattering in an ideal lattice.^35,49^ Additional scattering processes present under experimental conditions, including other phonon modes, charged impurities, structural defects, interfaces, and substrate effects, can reduce the experimentally attainable mobility.^49,50^ Furthermore, directional differences in electron and hole mobilities indicate anisotropic carrier transport but do not, by themselves, demonstrate spatial separation of photogenerated electrons and holes. In the present heterostructure, the proposed Z-scheme-like charge-separation behavior is instead supported by the interfacial charge redistribution, built-in electric field, band alignment, and band-resolved electronic states.

### 3.6 Gibbs free energy calculations for OER and HER

The reaction thermodynamics of the hydrogen evolution reaction (HER) and oxygen evolution reaction (OER) on the ZnI_2_/GeTe heterostructure were analyzed within the computational hydrogen electrode (CHE) framework at 298.15 K.^36^ The Gibbs free-energy change of an elementary electrochemical step was obtained from the DFT electronic-energy difference together with zero-point-energy and entropic corrections, while the electrode-potential contribution was treated consistently within the CHE convention:

9 Δ*G* = Δ*E* + ΔZPE − *T*Δ*S* + Δ*G*_U_ + Δ*G*_pH_

Here, Δ*E* is the electronic-energy difference between products and reactants, ΔZPE is the zero-point-energy correction, and Δ*S* is the entropy change. The detailed vibrational corrections and thermochemical data are provided in the SI. For a consistent thermodynamic comparison, the free-energy profiles discussed below are reported at pH = 0, where the SHE and RHE reference scales coincide. The photogenerated electron and hole potentials (*U*_e_ and *U*_h_) obtained from the band-edge positions are considered separately as the available reduction and oxidation driving forces and are not introduced as additional electrochemical potentials in the CHE free-energy expressions.

Consistent with the retained-carrier band alignment, OER was modeled on the I-exposed ZnI_2_ side and HER on the Ge-exposed side. OER intermediates OH*, O*, and OOH* were treated in a 2 × 2 ZnI_2_/GeTe supercell with one intermediate per supercell, corresponding to a nominal coverage of 0.25 relative to one adsorption site per 1 × 1 surface cell. The optimized O* configuration is approximately atop I, OH* lies near a surface-I site, and OOH* adopts a surface-bound configuration; optimized structures and relaxed bond distances are provided in the SI. The substrate atoms were kept fixed at the optimized heterostructure geometry while the adsorbate atoms were allowed to relax. OER was described by the conventional four proton-coupled electron-transfer steps:

10 * + H_2_O → OH* + H^+^ + e^−^

11 OH* → O* + H^+^ + e^−^

12 O* + H_2_O → OOH* + H^+^ + e^−^

13 OOH* → O_2_ + * + H^+^ + e^−^

At *U* = 0 V and pH = 0, the calculated free-energy changes for the four OER steps are 1.926, 0.972, 1.707, and 0.314 eV, respectively, giving an overall free-energy change of approximately 4.92 eV. The formation of OH* has the largest free-energy requirement and is therefore the potential-determining step. The corresponding limiting potential is obtained from14
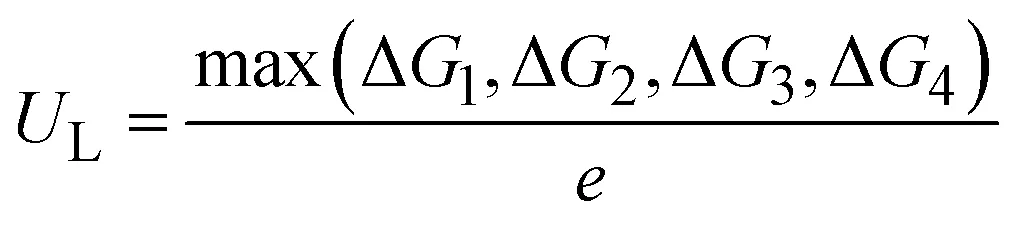
which gives a limiting potential of 1.926 V. The theoretical OER overpotential is therefore

15 *η*_OER_ = *U*_L_ − 1.23 V = 0.696 V ≈ 0.70 V

At the equilibrium OER potential *U* = 1.23 V, the four elementary free-energy changes become 0.696, −0.258, 0.477, and −0.916 eV, respectively. OH* formation remains the largest uphill step, giving a limiting potential of 1.926 V and a thermodynamic OER overpotential of approximately 0.70 V. For HER, three H-adsorption configurations were evaluated in the same 2 × 2 supercell with one H atom per supercell (nominal coverage 0.25). H1 and H3 are two inequivalent Ge-top configurations with optimized Ge–H distances of approximately 1.58 Å, whereas H2 is a Ge–Ge bridge configuration with two nearly equal Ge–H distances of approximately 1.92 Å. At pH = 0 and *U* = 0 V, the H-adsorption free energy was calculated from

16 Δ*G*(H*) = Δ*E*(H*) + ΔZPE − *T*Δ*S*

The calculated Δ*G*(H*) values are 0.360, 0.453, and 0.360 eV for H1, H2, and H3, respectively. H1 and H3 are therefore the most favorable among the examined configurations. An ideal HER descriptor is close to thermoneutral adsorption, Δ*G*(H*) ≈ 0 eV, because both H* formation and subsequent H_2_ release must remain accessible; increasingly negative adsorption free energy is therefore not intrinsically better. The optimized H1–H3 structures, H-related vibrational frequencies, ZPE values, ΔZPE corrections, and entropy contributions are provided in the SI. The CHE analysis and band-edge driving-force analysis describe complementary aspects of photocatalysis: CHE quantifies the thermodynamics of individual surface steps, whereas the band-edge analysis tests whether the retained GeTe electrons and ZnI_2_ holes possess sufficient reduction and oxidation power (Fig. 7).

**Fig. 7:**
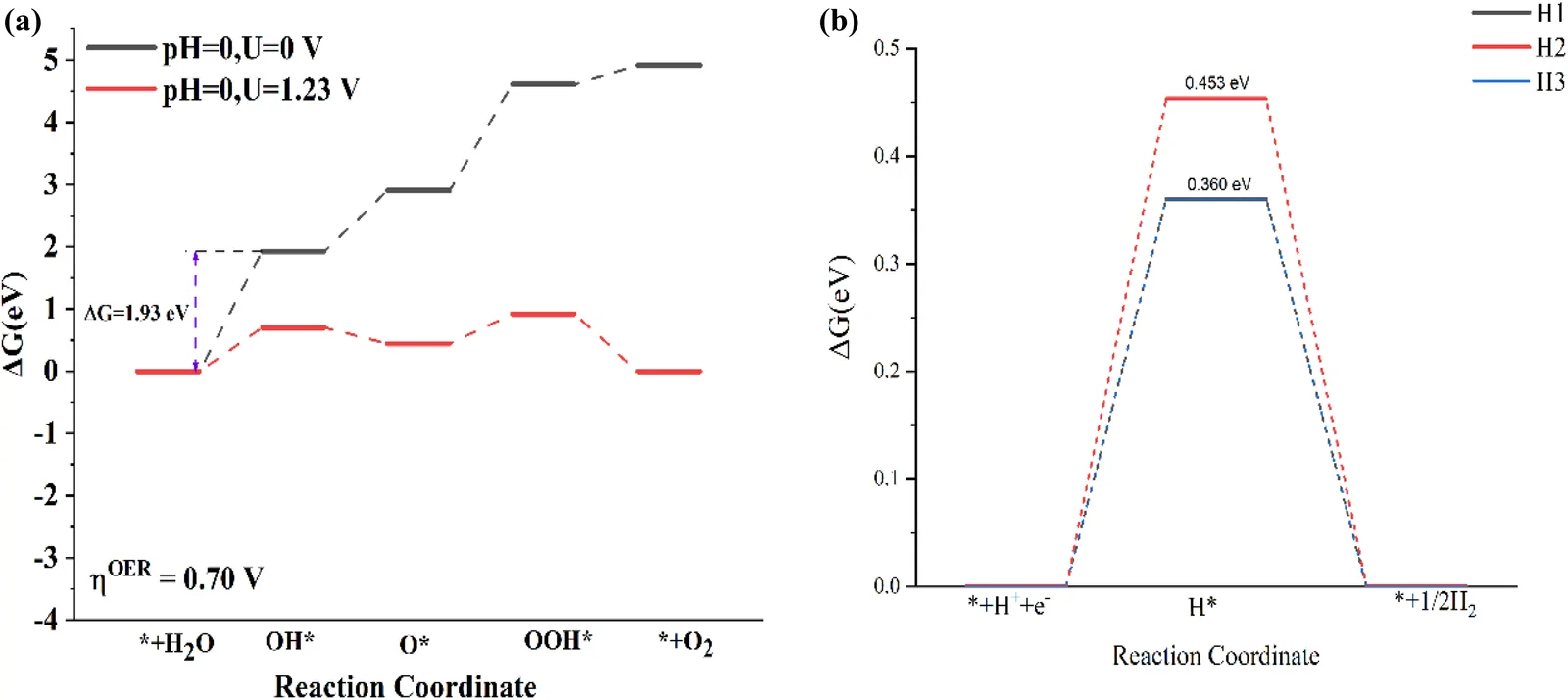
Gibbs free-energy profiles for water splitting on ZnI_2_/GeTe: (a) OER at pH = 0 and *U* = 0 and 1.23 V; (b) HER for H_1_–H_3_ at pH = 0 and *U* = 0 V. H_1_ and H_3_ overlap at Δ*G*(H*) = 0.360 eV, while H_2_ gives 0.453 eV. Dashed connecting lines are guides to the eye and do not represent kinetic activation barriers.

### 3.7 Optical absorption and solar-to-hydrogen (STH) efficiency

The optical response of the ZnI_2_ and GeTe monolayers and the ZnI_2_/GeTe heterostructure was evaluated from the frequency-dependent complex dielectric function. The absorption coefficient *α*(*ω*) was calculated as17
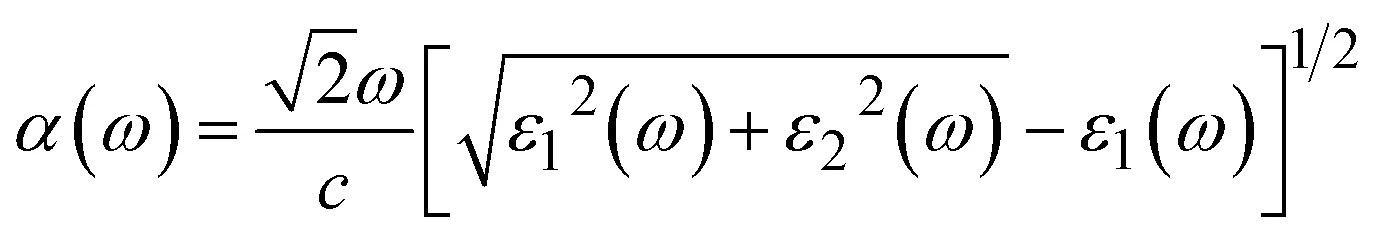
where *ω* is the angular frequency of the incident light, *c* is the speed of light, and *ε*_1_(*ω*) and *ε*_2_(*ω*) are the real and imaginary parts of the dielectric function, respectively.

Fig. 8 compares the calculated absorption spectra with the AM1.5G solar flux. Formation of the heterostructure produces a pronounced red shift relative to the isolated monolayers and enhances absorption over a broader part of the solar-energy range. This broader optical response is favorable for solar harvesting; however, optical absorption alone does not determine the fraction of absorbed photons that can drive the complete Z-scheme water-splitting process.

**Fig. 8:**
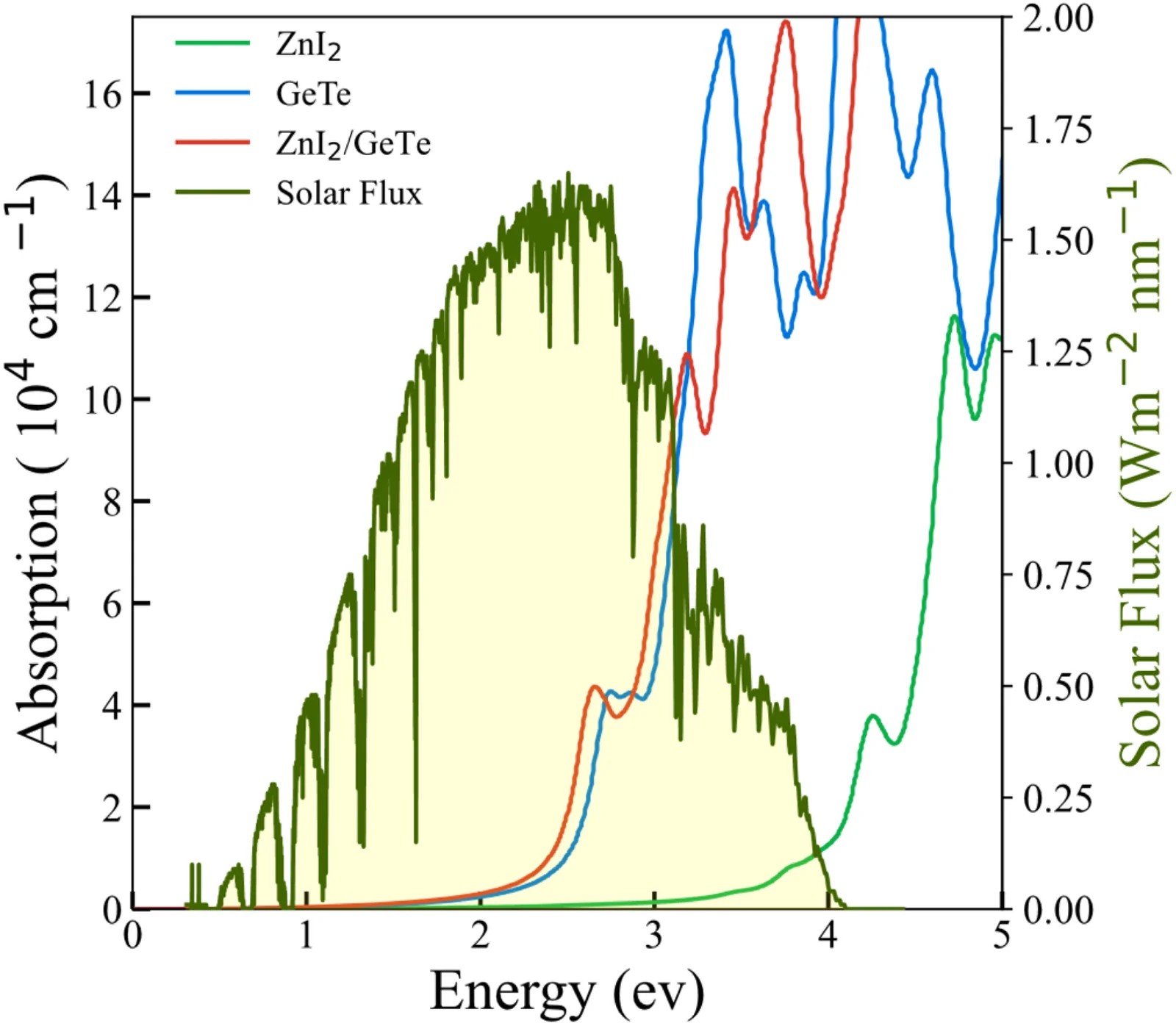
Optical absorption spectra of ZnI_2_, GeTe, and ZnI_2_/GeTe together with the AM1.5G solar flux.

The theoretical STH efficiency was evaluated using the direct Z-scheme formulation implemented in PySTH.^37^ In this framework, a factor of 1/2 accounts for the intrinsic carrier-utilization constraint of a direct Z-scheme: one photogenerated electron–hole pair is consumed through the interfacial Z-path while the complementary strongly reducing electron and strongly oxidizing hole remain available for HER and OER. The light-absorption and carrier-utilization terms were integrated over the AM1.5G spectral irradiance as defined below.18
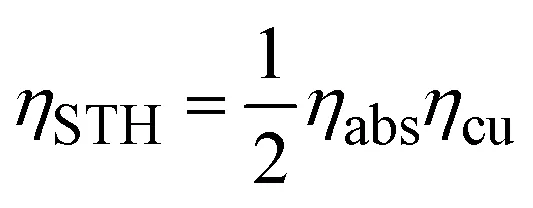
where the factor 1/2 accounts for the intrinsic carrier-utilization constraint of a direct Z-scheme: one set of photogenerated electrons and holes recombines through the interfacial Z-path, while the remaining strongly reducing electrons and strongly oxidizing holes participate in HER and OER, respectively. The two efficiency terms were obtained from the AM1.5 G spectral irradiance *P*(*E*) according to19
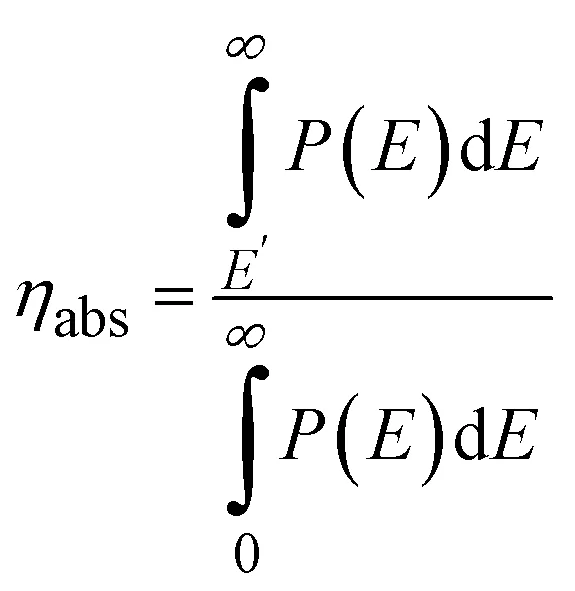
20
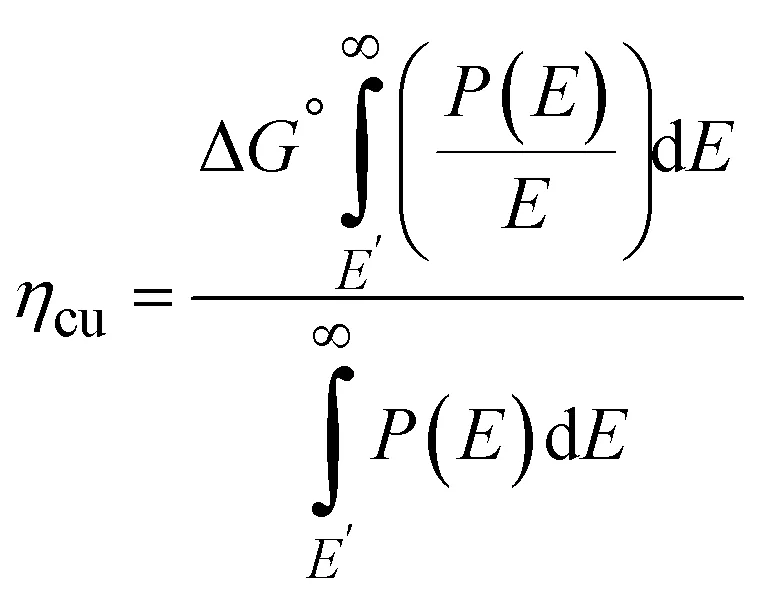
Here, *E* is the photon energy, Δ*G*° = 1.23 eV is the minimum thermodynamic free-energy requirement per transferred electron for overall water splitting, and *E*′ is the effective photon-energy threshold. For a direct Z-scheme heterostructure, productive photoexcitation is primarily associated with the individual semiconductor components rather than being assumed to arise from a strong direct interlayer transition. Accordingly, the relevant HSE06 gap entering the Z-scheme threshold was taken as the larger constituent band gap, consistent with recent treatments of Z-scheme photocatalysts:^44,45^

21 *E*^Z^_g_ = max(*E*^ZnI^_g_^_2_^, *E*^GeTe^_g_) = max(2.99, 2.16) = 2.99 eV

The reduced HSE06 electronic gap of the assembled heterostructure (1.96 eV) was not used as the productive photon threshold because photoexcitation is treated as occurring primarily within the individual constituents rather than through an assumed strong direct interlayer transition. The HSE06 gaps are 2.99 eV for ZnI_2_ and 2.16 eV for GeTe, so the direct Z-scheme threshold is *E*′ = max(2.99, 2.16) = 2.99 eV.^37,44,45^ For the current vacuum-referenced band-alignment dataset, *χ*(H_2_) ≈ 1.24 eV and *χ*(O_2_) ≈ 0.87 eV at pH = 0. Both exceed the PySTH branch criteria of 0.20 and 0.60 eV, respectively, so no additional HER/OER energy requirement is introduced.

22 *E*′ = *E*^Z^_g_ = 2.99 eV

The quantities *χ*(H_2_) and *χ*(O_2_) are band-edge redox driving forces and are not the same as the surface-reaction descriptors obtained from CHE. In particular, they are distinct from the OER overpotential and Δ*G*(H*). Because ZnI_2_ and GeTe are not Janus monolayers with an intrinsic out-of-plane vacuum-level difference, the Janus-specific intrinsic-field correction is not applied.

Integration of the AM1.5G spectrum with *E*′ = 2.99 eV gives a light-absorption efficiency *η*_abs_ = 6.32% and a carrier-utilization efficiency *η*_cu_ = 37.26%. Hence *η*_STH_ = 0.5 × 0.0632 × 0.3726 = 0.01178, corresponding to approximately 1.18%. The complete worked evaluation is also summarized in the SI.23
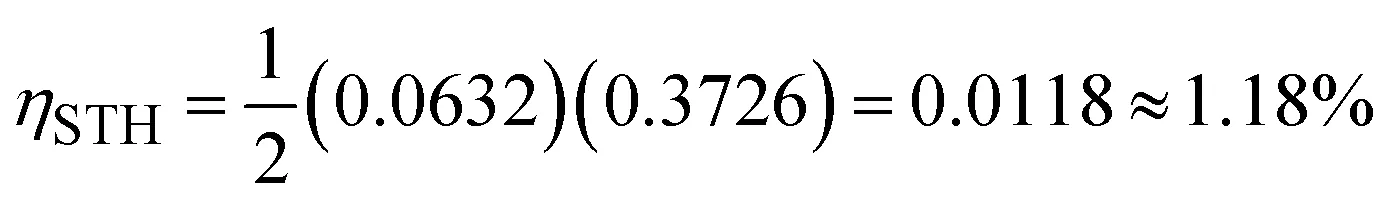


Thus, the predicted direct Z-scheme STH efficiency of ZnI_2_/GeTe is approximately 1.18%. This conservative value avoids using the reduced heterostructure gap as the productive excitation threshold and explicitly treats the AM1.5 G photon population, constituent-layer excitation thresholds, direct Z-scheme carrier utilization, and water-redox driving forces.

## 4 Conclusions

In conclusion, first-principles calculations identify AB-stacked ZnI_2_/GeTe as an energetically favorable vdW heterostructure for photocatalytic water splitting. The 3.27% isolated-layer mismatch is accommodated by a common in-plane lattice constant of 3.981 Å, giving residual strains of approximately −1.22% in ZnI_2_ and +2.07% in GeTe and a total strain energy of only 19.45 meV per primitive heterostructure cell. Among the five stackings, AB has the shortest interlayer distance (3.40 Å) and the most negative binding energy (−14.91 meV Å^−2^). The 8 ps AIMD simulation at 300 K retains the local Zn–I and Ge–Te coordination, with mean nearest-neighbor distances of 2.682 and 2.771 Å, respectively, and no persistent bond breaking or interfacial reconstruction. The equilibrium electronic structure is staggered type-II, but this static alignment is not used alone to assign the photoinduced pathway. Instead, the GeTe → ZnI_2_ ground-state charge transfer (∼0.037|*e*|), positive work-function mismatch, Δ*Φ* ≈ 0.27 eV interfacial potential step, vacuum-referenced band alignment, and band-decomposed charge densities collectively support a direct Z-scheme-like pathway while remaining short of a time-resolved proof. The heterostructure also exhibits anisotropic intrinsic carrier transport, an OER overpotential of approximately 0.70 V at pH = 0, H-adsorption free energies of 0.360–0.453 eV for the examined HER configurations, broadened optical absorption, and a direct Z-scheme STH efficiency of 1.18%.

## Conflicts of interest

There are no conflicts to declare.

## Supplementary Material

RA-016-D6RA06043A-s001

## Data Availability

The data supporting this article have been included as part of the supplementary information (SI). Supplementary information is available. See DOI: https://doi.org/10.1039/d6ra06043a.
