# Supplementary material for: Direct Z-scheme ZnI2/GeTe van der Waals heterostructure for photocatalytic overall water splitting
Source: RSC Adv. 2026 Sep 4;16(43):52892–904. doi: 10.1039/d6ra06043a (PMC13542888; doi:10.1039/d6ra06043a)
Supplement: Supplementary file 1 [file RA-016-D6RA06043A-s001.pdf]

Supplementary Information (SI) for RSC Advances.
This journal is © The Royal Society of Chemistry 2026

Supplementary Materials

Direct Z-Scheme ZnI_2/GeTe Van Der Waals Heterostructure for Photocatalytic Overall Water Splitting

Muhmmad Kashif[a*], Aamir Shahzad[b], Ateeq-ur-Rehman[c*]

[a,b] Physics Department, Govt. College University Faisalabad 38000, Pakistan

[c] Physics Department, University of Agriculture, Faisalabad 38040, Pakistan

Corresponding Authors: mkashif@gcuf.edu.pk, ateeq.rehman@uaf.edu.pk

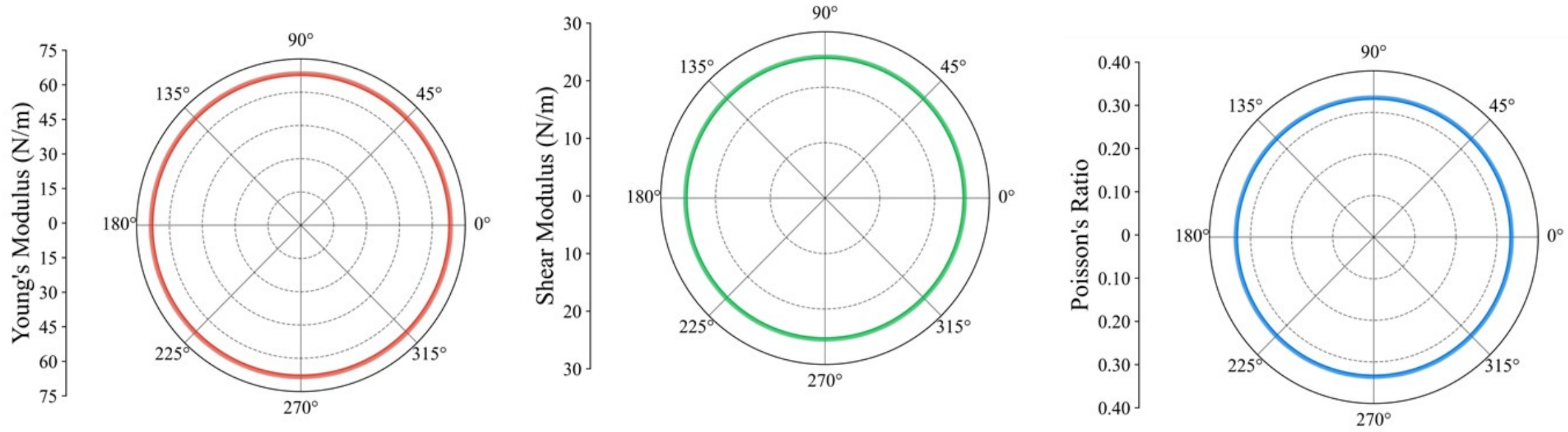

Fig.S1 Two-dimensional polar plots of Young's modulus, shear modulus, and Poisson's ratio of the ZnI_2/GeTe heterostructure.

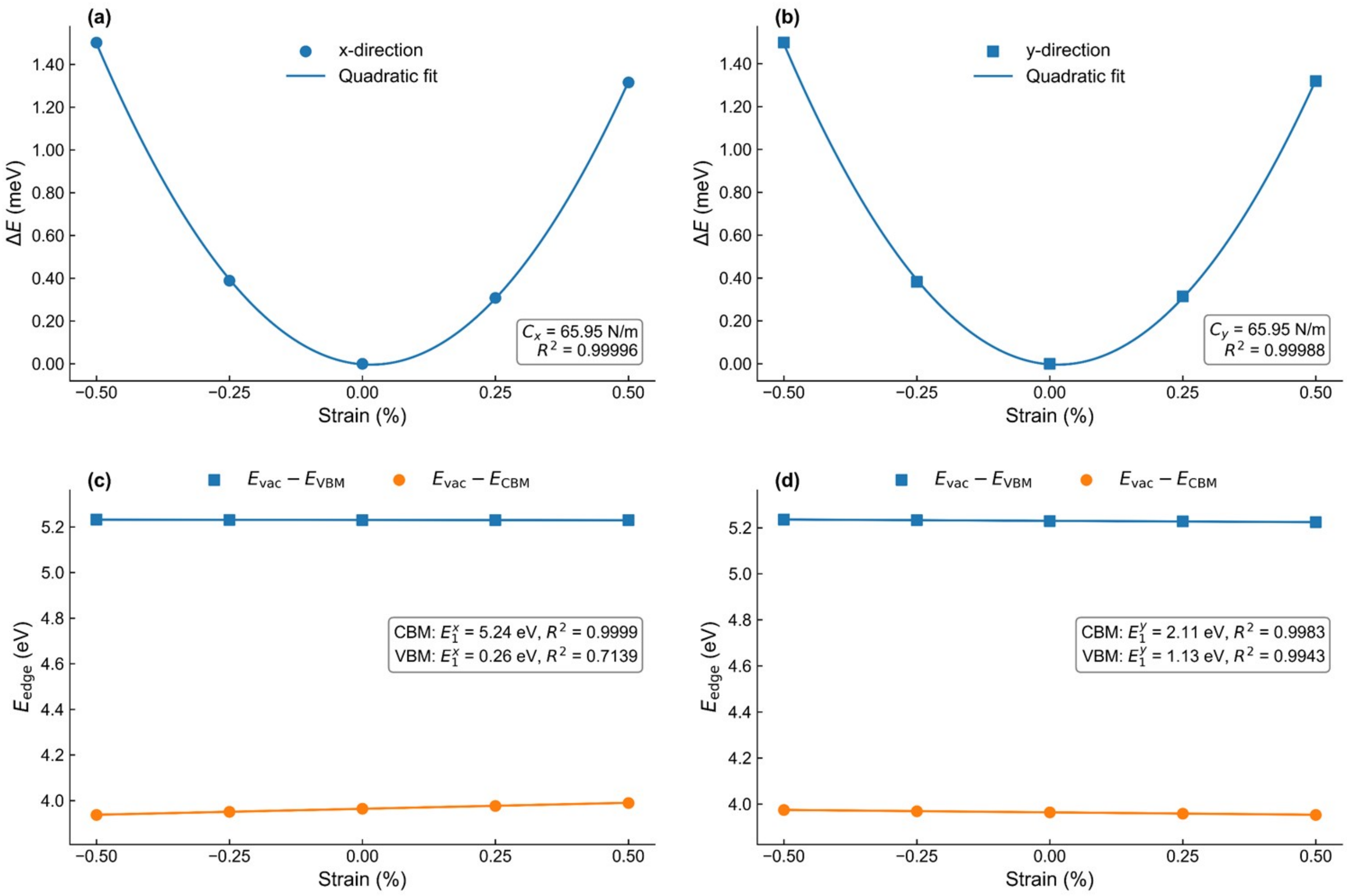

Fig.S2 (a,b)total energy and (c,d) band edge positions of ZnI_2/GeTe heterostructure as a function of the uniaxial strain ε along both the x and y directions

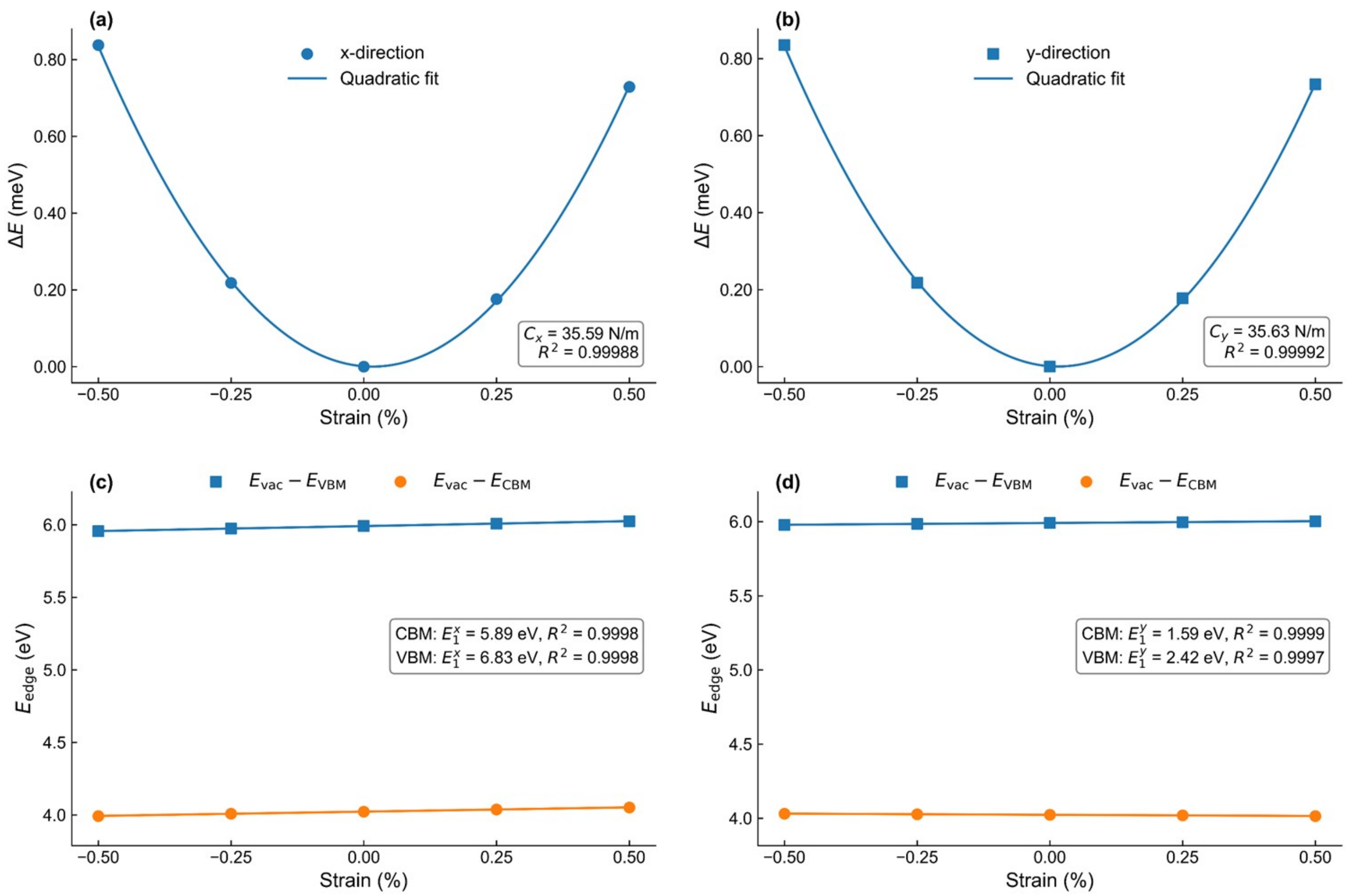

Fig.S3 (a,b)total energy and (c,d) band edge positions of ZnI_2 monolayer as a function of the uniaxial strain along both the x and y directions

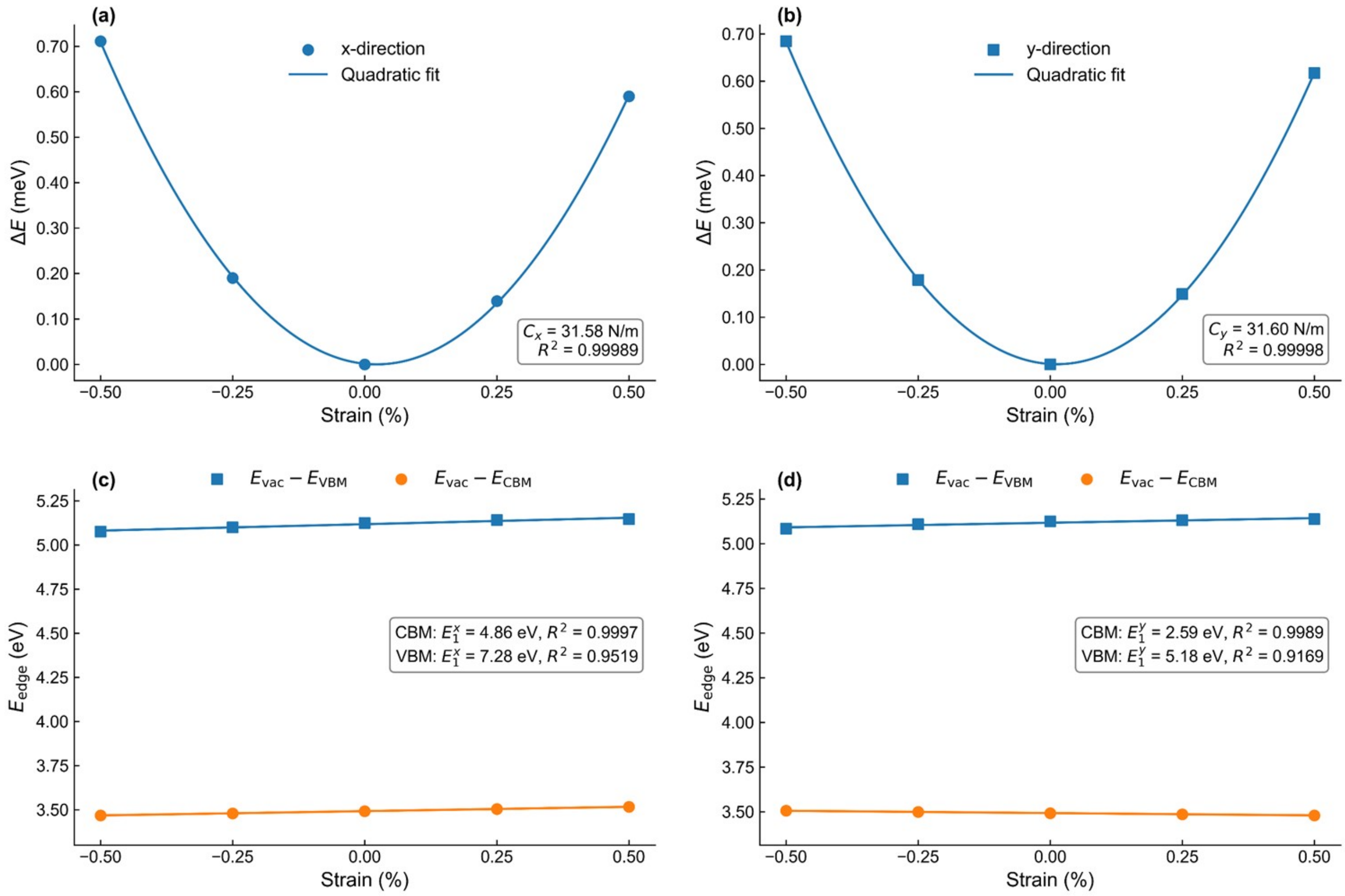

Fig.S4 (a,b)total energy and (c,d) band edge positions of GeTe monolayer as a function of the uniaxial strain along both the x and y directions

Gibbs Free-Energy Calculations for HER and OER

Catalytic models and optimized intermediates

The surface reaction models were constructed using a 2×2 ZnI_2/GeTe heterostructure supercell (a = b = 7.9594 Å and c = 24.5238 Å). For OER, one O*, OH*, or OOH* intermediate was placed on the I-exposed ZnI_2 side per 2×2 supercell. During optimization of these adsorbed intermediates, the underlying heterostructure was kept at its optimized geometry, whereas the adsorbate atoms were allowed to relax without positional constraints. The optimized side and top views are shown in Fig. S5. The principal relaxed bond distances and adsorption geometries are summarized in Table S1.

Table S1a. Structural details of the optimized OER intermediates on the 2×2 ZnI_2/GeTe heterostructure.

Intermediate	Surface / model	Adsorbate per cell	Optimized adsorption geometry	Key relaxed distance(s) (Å)	Relaxation
O*	I-exposed ZnI_2 side; 2×2 supercell	1 O	Approximately atop-I	O-I = 1.856	Adsorbate relaxed; substrate fixed
OH*	I-exposed ZnI_2 side; 2×2 supercell	1 OH	Near surface-I site	O-H = 0.981; nearest O-I = 2.260	Adsorbate relaxed; substrate fixed
OOH*	I-exposed ZnI_2 side; 2×2 supercell	1 OOH	Surface-bound OOH configuration	O-O = 1.380; O-H = 0.994; nearest O-I = 2.739, 2.761	Adsorbate relaxed; substrate fixed

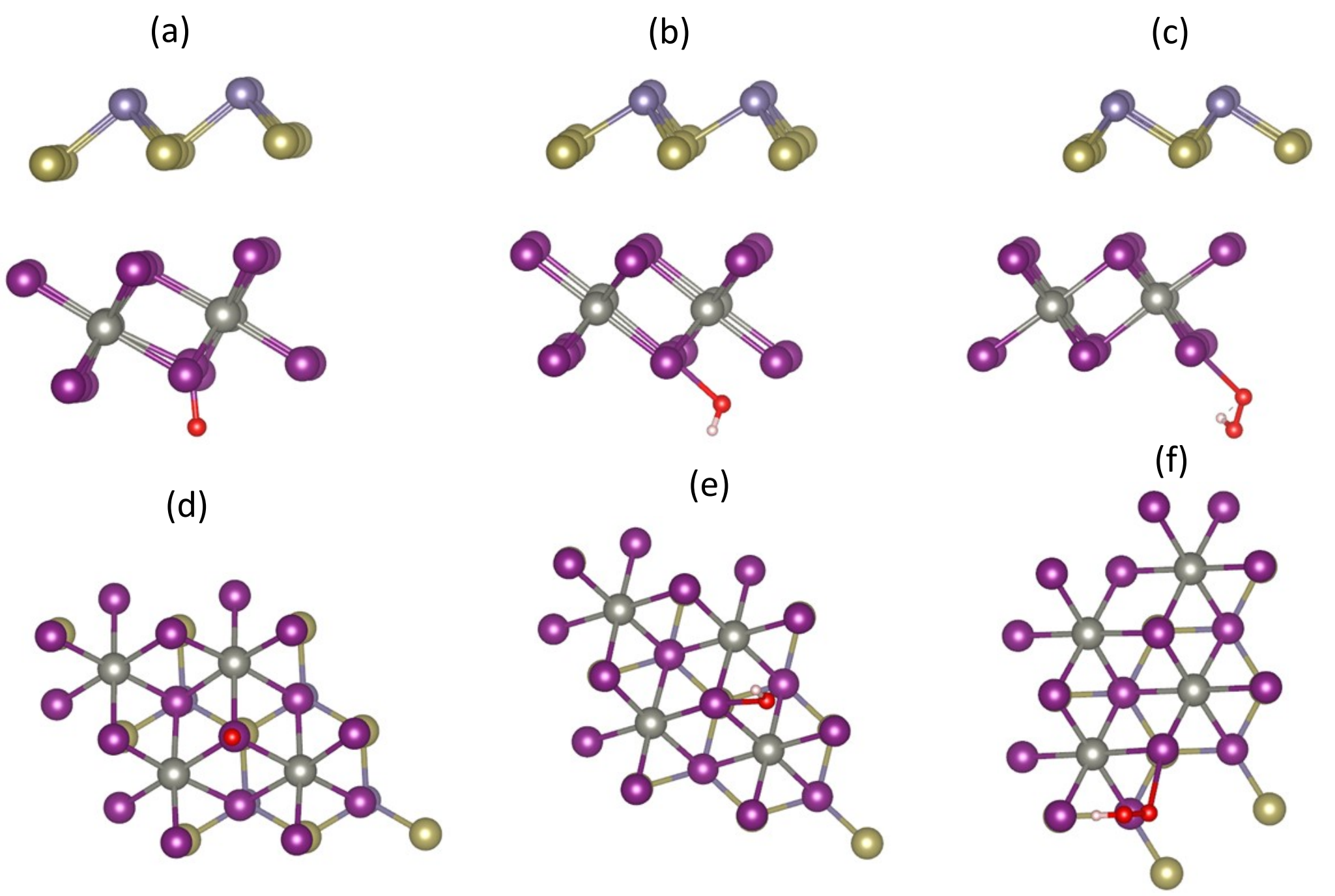

Fig.S5 Side and top views of the optimized (a,d) O*, (b,e) OH*, and (c,f) OOH*adsorption configurations on the I-exposed ZnI_2 side of the 2×2 ZnI_2/GeTe heterostructure. One reaction intermediate was included per 2×2 supercell. Only one periodic image of each adsorbate is displayed for clarity.

For HER, three hydrogen adsorption configurations, denoted H_1, H_2, and H_3, were evaluated on the Ge-exposed side of the same 2 × 2 ZnI_2/GeTe supercell. H_1 and H_3 correspond to two inequivalent Ge-top configurations with optimized Ge–H distances of approximately 1.580 and 1.580 Å, respectively, whereas H_2 is a Ge–Ge bridge configuration with two nearly equal Ge–H distances of approximately 1.920 Å. One H atom was included per 2 × 2 supercell (nominal coverage 0.25 H per primitive surface cell). The optimized top and side views are shown in Fig. S6, and the principal structural and vibrational data are summarized in Table S1b.

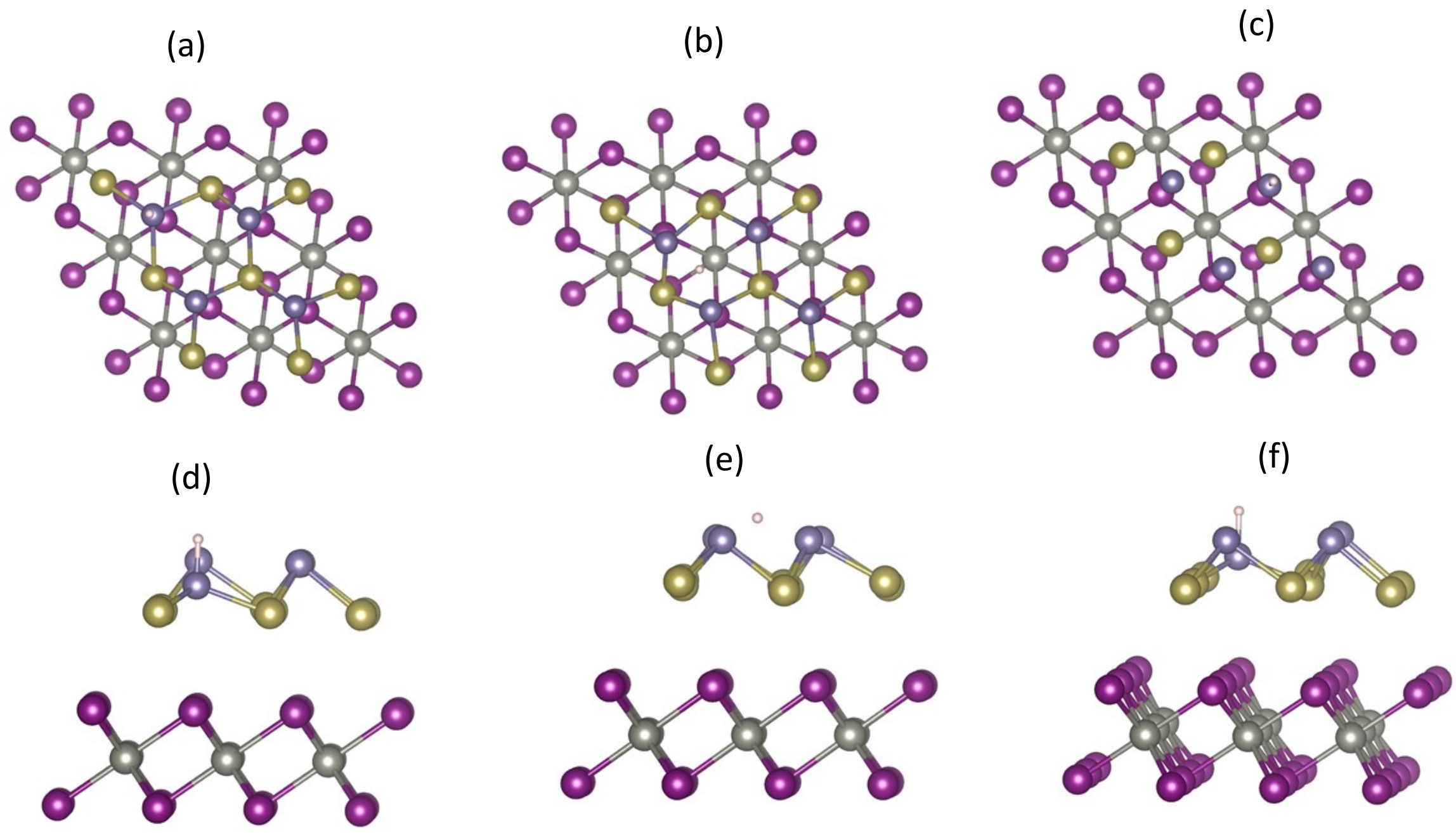

Fig. S6 Top and side views of the optimized H_1, H_2, and H_3 adsorption configurations on the Ge-exposed side of the 2 × 2 ZnI_2/GeTe heterostructure. H_1 and H_3 are inequivalent Ge-top configurations, while H_2 is a Ge-Ge bridge configuration.

Table S1b. Structural and H-related vibrational data for the optimized HER adsorption configurations.

**Configuration**	**Optimized site**	**Key Ge-H distance(s) (Å)**	**H vibrational frequencies (cm^{-1})**	**ZPE(H*) (eV)**	**ΔZPE (eV)**
H_1	Ge-top	1.580	1813.60, 474.13, 454.88	0.170020	+0.035765
H_2	Ge-Ge bridge	1.920, 1.920	853.12, 724.99, 524.03	0.130316	−0.003955
H_3	Ge-top	1.580	1814.23, 474.77, 464.17	0.170675	+0.036418

Vibrational thermochemical corrections

For the OER adsorbates, vibrational thermochemical corrections were obtained with VASPKIT at 298.15 K. In the VASPKIT treatment used here, only vibrational contributions are included for adsorbed species; translational, rotational, and electronic contributions are neglected, and frequencies below 50 cm^{-1} are set to 50 cm^{-1} to avoid unrealistically large entropy contributions from low-frequency modes.

$$H_{corr}(T) = E_{ZPE} + \Delta U(0 \rightarrow T) \tag{S1}$$

$$G_{corr}(T) = H_{corr}(T) - TS = E_{ZPE} + \Delta U(0 \rightarrow T) - TS \tag{S2}$$

$$G = E_{DFT} + G_{corr}(T) \tag{S3}$$

Here E_{ZPE} is the zero-point energy, ΔU(0→T) is the vibrational thermal-energy contribution from 0 K to T, TS is the vibrational entropy contribution, and $G_{corr(T)}$ is the thermal Gibbs correction added to the DFT electronic energy. The numerical VASPKIT values used for OH*, O*, and OOH* are listed in Table S3.

Computational hydrogen electrode and potential/pH convention

The electrochemical free energies were described within the computational hydrogen electrode (CHE) framework, following the standard DFT-based treatment of proton-coupled electron-transfer reactions. The reaction free energy is written in terms of the DFT energy difference, the vibrational/thermal correction, and the electrochemical potential and pH contributions. [1,3,4]

$$\Delta G = \Delta E_{DFT} + \Delta G_{vib} + \Delta G_U + \Delta G_{pH} \tag{S4}$$

$$\alpha = k_B T ln(10) = 0.05916\ eV \quad (T = 298.15\ K) \tag{S5}$$

$$\Delta G = \Delta E_{DFT} + \Delta G_{vib} + \Delta G_{U} + \Delta G_{pH} \quad \text{(S4)}$$

$$\mu_{pe} = \frac{1}{2}G_{H_2} - eU_{SHE} - \alpha\, pH \quad \text{(S6)}$$

$$U_{RHE} = U_{SHE} + 0.05916\, pH \quad \text{(S7)}$$

$$\mu_{pe} = \frac{1}{2}G_{H_2} - eU_{RHE} \quad \text{(S8)}$$

Equations S5-S8 also show the distinction between SHE and RHE referencing. When U is expressed versus RHE, the pH dependence is already incorporated into the reference potential and an additional 0.05916×pH term must not be added again. This convention avoids double counting of the pH contribution.

Hydrogen evolution reaction

Hydrogen adsorption was described by the Volmer-type proton-electron transfer step. In Eqs. S9-S18, S denotes a vacant surface site and the subscript ads corresponds to the conventional H*, OH*, O*, and OOH* adsorbate notation.

$$S + H^{+} + e^{-} \rightarrow H_{ads} \quad \text{(S9)}$$

$$\Delta E_{H_{ads}} = E_{slab+H} - E_{slab} - \frac{1}{2}E_{H_2} \quad \text{(S10)}$$

$$\Delta G_{H_{ads}} = \Delta E_{H_{ads}} + \Delta ZPE - T\Delta S \quad \text{(S11)}$$

$$\Delta G_{H_{ads}}(U,pH) = \Delta G_{H_{ads}}(0,0) + eU_{SHE} + \alpha\, pH \quad \text{(S12)}$$

$$\Delta G_{H_{ads}}(U_{RHE}) = \Delta G_{H_{ads}}(0,0) + eU_{RHE} \quad \text{(S13)}$$

The molecular-hydrogen reference quantities used were $E(H_2)$ = -6.773557 eV, $1/2E(H_2)$ = -3.3867785 eV, $ZPE(H_2)$ = 0.268513 eV, $1/2ZPE(H_2)$ = 0.1342565 eV, and $TS(H_2)$ = 0.404038 eV at 298.15 K. The site-resolved corrections and final adsorption free energies are summarized in Table S2.

$$\Delta G_{H_{ads}}(H1) = 0.359513\ eV \approx 0.360\ eV \quad \text{(S14)}$$

The corresponding electronic adsorption energies, ZPE corrections, entropy terms, and final ΔG(H*) values are listed below. The molecular H_2 reference is specified immediately above.

H configuration	ΔE (H*) (eV)	ΔZPE (eV)	-TΔS (eV)	ΔG(H*) (eV)
H_1	0.141256	0.035765	0.182492	0.359513 ≈ 0.360
H_2	0.268770	-0.003955	0.188486	0.453301 ≈ 0.453
H_3	0.141183	0.036418	0.182866	0.360467 ≈ 0.360

Table S2. Site-resolved HER thermochemical quantities at pH = 0 and U = 0 V.

H_1 and H_3 therefore give the lowest ΔG_{H^*} among the three investigated configurations. The ideal HER descriptor is Δ_{GH^*} close to 0 eV: excessively weak H binding makes H* formation unfavorable, whereas excessively strong binding can hinder subsequent H_2 release. Thus, increasingly negative ΔG_{H^*} should not be interpreted as continuously improving HER activity. [2]

Oxygen evolution reaction

The OER was represented by the conventional four-step proton-coupled electron-transfer pathway involving OH*, O*, and OOH* intermediates. [3,4]

$$S + H_2O \rightarrow OH_{ads} + H^+ + e^- \quad \text{(S15a)}$$

$$OH_{ads} \rightarrow O_{ads} + H^+ + e^- \quad \text{(S15b)}$$

$$O_{ads} + H_2O \rightarrow OOH_{ads} + H^+ + e^- \quad \text{(S15c)}$$

$$OOH_{ads} \rightarrow O_2 + S + H^+ + e^- \quad \text{(S15d)}$$

Table S3. VASPKIT vibrational thermochemical corrections for the OER intermediates at 298.15 K.

Intermediate	E_{DFT} (eV)	E_{ZPE} (eV)	ΔU(0→T) (eV)	TS (eV)	G_{corr}(T) (eV)	$E_{DFT} + G_{corr}$ (eV)
OH*	-66.794978	0.305867	0.076656	0.164020	0.218503	-66.576475

Intermediate	E_{DFT} (eV)	E_{ZPE} (eV)	ΔU(0→T) (eV)	TS (eV)	G_{corr}(T) (eV)	E_{DFT} + G_{corr} (eV)
O*	-62.178773	0.050660	0.046672	0.112961	-0.015629	-62.194402
OOH*	-71.580289	0.414068	0.106114	0.237303	0.282880	-71.297409

The corrected Gibbs energies of the adsorbed intermediates were combined with the corresponding clean-slab and molecular reference energies to construct the OER reaction free-energy profile. At pH = 0 and U = 0 V, the four step free energies are ΔG_1 = 1.926139 eV, ΔG_2 = 0.972493 eV, ΔG_3 = 1.707414 eV, and ΔG_4 = 0.313954 eV.

$$\sum_{i=1}^{4} \Delta G_i = 4.920000\ eV \tag{S16}$$

$$\Delta G_i(U,pH) = \Delta G_i(0,0) - eU_{SHE} - \alpha\, pH \tag{S17}$$

$$\Delta G_i(U_{RHE}) = \Delta G_i(0,0) - eU_{RHE} \tag{S18}$$

$$U_L = \frac{max(\Delta G_1, \Delta G_2, \Delta G_3, \Delta G_4)}{e} = 1.926\ V \tag{S19}$$

$$\eta_{OER} = U_L - 1.23 = 1.926 - 1.23 = 0.696\ V \approx 0.70\ V \tag{S20}$$

Table S4. OER step free energies at pH = 0 under U = 0 and 1.23 V.

Step	Elementary reaction	ΔG at U = 0 V (eV)	ΔG at U = 1.23 V (eV)
1	* + H_2O → OH* + H^+ + e^-	1.926139	0.696139
2	OH* → O* + H^+ + e^-	0.972493	-0.257507
3	O* + H_2O → OOH* + H^+ + e^-	1.707414	0.477414
4	OOH* → O_2 + * + H^+ + e^-	0.313954	-0.916046

The OH* formation step has the largest free-energy requirement and is therefore the potential-determining step. The corresponding limiting potential is U_L = 1.926 V and the theoretical thermodynamic OER overpotential is η_{OER} = 0.696 V (approximately 0.70 V). At the equilibrium OER potential of 1.23 V, OH* formation remains uphill by 0.696 eV and OOH* formation remains uphill by 0.477 eV. The CHE free-energy analysis is thermodynamic and, by itself, does not establish faster or slower OER kinetics at different pH values.

Photogenerated-carrier driving forces

The band-edge-derived photogenerated electron and hole potentials, U_e and U_h, are considered separately from the electrochemical potential U used in the CHE expressions. U_e and U_h describe the energetic reduction and oxidation driving forces supplied by the surviving photogenerated carriers, whereas the CHE treatment describes the thermodynamics of the individual surface reaction steps. Closely related first-principles photocatalytic studies use this separation when comparing carrier driving forces with HER/OER reaction energetics. [5-7]

Accordingly, U_e and U_h are not introduced as additional electrode-potential corrections in Eqs. S12, S13, S17, or S18. This avoids mixing pH-dependent water-redox references with independently shifted photogenerated-carrier energies.

Additional band-alignment, mobility, and STH reproducibility data, Vacuum-referenced band alignment. The numerical energies used in the interfacial band-alignment diagram are summarized in Table S5. Work functions are reported with the standard positive convention

$\Phi = E_{vac} - E_F$.

Table S5. Vacuum-referenced electronic levels and work-function quantities used in the band-alignment analysis.

**Quantity**	**ZnI_2**	**GeTe**	**Notes**
Isolated CBM (eV)	−3.67	−3.26	Before contact
Isolated VBM (eV)	−6.66	−5.43	Before contact
Isolated work function Φ (eV)	6.57	5.38	Positive convention

Quantity	ZnI_2	GeTe	Notes
Layer-resolved CBM after contact (eV)	−3.69	−3.20	Common vacuum reference
Layer-resolved VBM after contact (eV)	−6.54	−5.66	Common vacuum reference
Surface work-function magnitude after contact (eV)	5.60	5.33	$\Delta\Phi \approx 0.27$ eV

At pH = 0, the after-contact GeTe-derived CBM at approximately −3.20 eV gives $\chi(H_2) = (-3.20) - (-4.44) \approx 1.24$ eV, while the ZnI_2-derived VBM at approximately −6.54 eV gives $\chi(O_2) = (-5.67) - (-6.54) \approx 0.87$ eV. These are the vacuum-referenced redox-driving-force values used in the final PySTH evaluation[8].

Carrier-mobility reproducibility. The x and y labels denote the two in-plane lattice-vector directions. Effective masses were evaluated at the actual electron and hole extrema. The deformation-potential fits used the same ±0.5% strain range shown in Figs. S2-S4. Table S6 collects the extrema, directional masses, and deformation-potential fit quality.

Table S6. Carrier extrema, effective masses, and deformation-potential fit quality used for the mobility calculations.

System / carrier	Band extremum	m^*_x/m_0	m^*_y/m_0	$R^2(E_{1x})$	$R^2(E_{1y})$
ZnI_2 electron	Γ	0.276	0.630	0.9998	0.9999
ZnI_2 hole	off-Γ VBM near (0, 0.217172, 0)	0.261	1.681	0.9998	0.9997
GeTe electron	off-Γ CBM near (0, 0.378788, 0)	0.180	0.464	0.9997	0.9989
GeTe hole	Γ	0.912	0.531	0.9519	0.9169
ZnI_2/GeTe electron	Γ	0.253	0.640	0.9999	0.9983
ZnI_2/GeTe hole	VBM at (0.166667, 0, 0)	1.449	2.357	0.7139	0.9943

Effective masses were obtained from the local parabolic curvature at the actual carrier extrema. Table S6 reports the extrema and directional masses, while Figs. S2–S4 document the strain-energy and vacuum-referenced band-edge fits used to obtain the elastic and deformation-potential parameters. This presentation avoids assigning a common high-symmetry extremum to carriers whose band extrema occur off Γ.

Solar-to-hydrogen efficiency verification. Productive excitation was treated as primarily intralayer; therefore the larger constituent HSE06 gap, rather than the reduced 1.96 eV heterostructure gap, was used as the direct Z-scheme photon threshold. For ZnI_2/GeTe, E′ = max(2.99, 2.16) = 2.99 eV. The PySTH branch conditions are satisfied because the retained-carrier HER and OER driving forces exceed 0.20 and 0.60 eV, respectively. Integration of the AM1.5G spectrum gives ηabs = 6.32% and ηcu = 37.26%, so

$$\eta_{STH} = 0.5 \times \eta_{abs} \times \eta_{cu} = 0.5 \times 0.0632 \times 0.3726 = 0.01178 \approx 1.18\%.$$

Table S7. Numerical inputs and output of the direct Z-scheme STH evaluation.

**Quantity**	**Value**
ZnI_2 HSE06 gap	2.99 eV
GeTe HSE06 gap	2.16 eV
Effective threshold E′	2.99 eV
$\chi(H_2)$, current figure/input	≈1.24 eV
$\chi(O_2)$, current figure/input	≈0.87 eV
PySTH branch thresholds	0.20 eV (HER); 0.60 eV (OER)
ηabs	6.32%
ηcu	37.26%
Direct Z-scheme prefactor	0.5
ηSTH	1.18%

AIMD production-trajectory and bonding verification

Finite-temperature structural integrity was assessed from a 3 × 3 ZnI_2/GeTe AIMD supercell (45 atoms) at 300 K in the NVT ensemble using a 1 fs time step for 8 ps. The mean temperature is 299.96 ± 8.35 K and Nearest-neighbor and interlayer metrics used to assess bond retention are summarized in Table S8.

Table S8. AIMD simulation and structural/bonding metrics for ZnI_2/GeTe at 300 K.

Metric	Value
AIMD supercell	3 × 3; 45 atoms ($Zn_9I_{18}Ge_9Te_9$)
Ensemble / target temperature / time step/ total time	NVT / 300 K / 1 fs/ 8 ps
Temperature	299.96 ± 8.35 K; range 274–329 K
Zn–I nearest-neighbor metric	2.6820 ± 0.0156 Å; first/final = 2.7048/2.6814 Å
Ge–Te nearest-neighbor metric	2.7708 ± 0.0119 Å; first/final = 2.7586/2.7760 Å
Minimum interlayer I–Te separation	3.5936 ± 0.0928 Å; minimum observed = 3.3011 Å
Final local coordination (3.2 Å cutoff)	All 9 Zn sites retain ≥4 I neighbors; all 9 Ge sites retain 3 Te neighbors
Structural observation	No persistent bond breaking or interfacial reconstruction

The Zn–I and Ge–Te distances fluctuate around stable mean values and recover comparable values at the end of the simulation window. The preserved final coordination and nonbonding interlayer I–Te separation show that the 300 K AIMD simulation does not produce persistent bond cleavage or interface reconstruction. These data support finite-temperature structural integrity over the analyzed 8 ps simulation window without implying longer-time kinetic stability.

References

1 J. K. Nørskov, J. Rossmeisl, A. Logadottir, L. Lindqvist, J. R. Kitchin, T. Bligaard and H. Jónsson, J. Phys. Chem. B, 2004, 108, 17886-17892. DOI: 10.1021/jp047349j.

2 J. K. Nørskov, T. Bligaard, A. Logadottir, J. R. Kitchin, J. G. Chen, S. Pandelov and U. Stimming, J. Electrochem. Soc., 2005, 152, J23-J26. DOI: 10.1149/1.1856988.

3 J. Rossmeisl, Z.-W. Qu, H. Zhu, G.-J. Kroes and J. K. Nørskov, J. Electroanal. Chem., 2007, 607, 83-89. DOI: 10.1016/j.jelechem.2006.11.008.

4 I. C. Man, H.-Y. Su, F. Calle-Vallejo, H. A. Hansen, J. I. Martínez, N. G. Inoglu, J. Kitchin, T. F. Jaramillo, J. K. Nørskov and J. Rossmeisl, ChemCatChem, 2011, 3, 1159-1165. DOI: 10.1002/cctc.201000397.

5 X.-Q. Wan, C.-L. Yang, X.-H. Li, Y.-L. Liu and W.-K. Zhao, J. Mater. Chem. A, 2024, 12, 2359-2372. DOI: 10.1039/D3TA06755F.

6 R. Sun, C.-L. Yang, M.-S. Wang and X.-G. Ma, J. Power Sources, 2022, 547, 232008. DOI: 10.1016/j.jpowsour.2022.232008.

7 X.-Q. Wan, C.-L. Yang, X.-H. Li, M.-S. Wang and X.-G. Ma, J. Phys. Chem. Lett., 2023, 14, 9126-9135. DOI: 10.1021/acs.jpclett.3c01780.

8 Q. Li, M. Tao, Y. Liu, H. Huang, Q. Li and X. Zhu, Comput. Phys. Commun., 2025, 317, 109822. DOI: 10.1016/j.cpc.2025.109822.
